# A fijiviral nonstructural protein triggers cell death in plant and bacterial cells via its transmembrane domain

**DOI:** 10.1111/mpp.13277

**Published:** 2022-10-28

**Authors:** Zhengjie Yuan, Yanfei Geng, Yuanxing Dai, Jing Li, Mingfang Lv, Qiansheng Liao, Li Xie, Heng‐Mu Zhang

**Affiliations:** ^1^ Laboratory of Virology, Institute of Virology and Biotechnology Zhejiang Academy of Agricultural Sciences Hangzhou China; ^2^ College of Chemistry and Life Science Zhejiang Normal University Jinhua China; ^3^ College of Life Science Zhejiang Sci‐Tech University Hangzhou China; ^4^ Analysis Center of Agrobiology and Environmental Sciences Zhejiang University Hangzhou China

**Keywords:** cell death, fijivirus, transmembrane domain, viral nonstructural protein

## Abstract

*Southern rice black‐streaked dwarf virus* (SRBSDV; *Fijivirus*, *Reoviridae*) has become a threat to cereal production in East Asia in recent years. Our previous cytopathologic studies have suggested that SRBSDV induces a process resembling programmed cell death in infected tissues that results in distinctive growth abnormalities. The viral product responsible for the cell death, however, remains unknown. Here P9‐2 protein, but not its RNA, was shown to induce cell death in *Escherichia coli* and plant cells when expressed either locally with a transient expression vector or systemically using a heterologous virus. Both computer prediction and fluorescent assays indicated that the viral nonstructural protein was targeted to the plasma membrane (PM) and further modification of its subcellular localization abolished its ability to induce cell death, indicating that its PM localization was required for the cell death induction. P9‐2 was predicted to harbour two transmembrane helices within its central hydrophobic domain. A series of mutation assays further showed that its central transmembrane hydrophobic domain was crucial for cell death induction and that its conserved F90, Y101, and L103 amino acid residues could play synergistic roles in maintaining its ability to induce cell death. Its homologues in other fijiviruses also induced cell death in plant and bacterial cells, implying that the fijiviral nonstructural protein may trigger cell death by targeting conserved cellular factors or via a highly conserved mechanism.

## Graphical abstract

Fijiviral P9‐2 protein but not RNA was shown to induce cell death by targeting the plasma membrane in plant cells, suggesting that this protein could be responsible for a programmed cell death‐like process in infected tissues with hyperplasia.

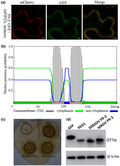

## 1 INTRODUCTION

Reoviruses are a large family of viruses that naturally infect a wide range of eukaryotes, including fungi, plants, and animals from insects to mammals (Attoui et al., 2012). Plant‐infecting reoviruses are classified into three genera, *Fijivirus*, *Oryzavirus*, and *Phytoreovirus*, and all known members of them are insect‐borne in a persistent manner. They can multiply in both their plant hosts and insect vectors, and cause serious symptoms in plant hosts but appear to be innocuous in insect vectors (Attoui et al., 2012). It is interesting that most plant‐infecting reoviruses are phloem‐limited and induce similar cytopathologic features, including phloem cell hyperplasia or hypertrophy, which can lead to swelling, galls, enation or tumours on the veins of host plants (Lv et al., 2017a). Histological studies have shown that plant reovirus‐induced tumours are highly organized and always contain three cell types: phloem parenchyma (PPs), sieve elements (SEs), and vessels (Xie et al., 2014). Within the tumour phloem, SEs, cells that are terminally enucleated and differentiated, show unexpected hyperplasia together with PPs and aggregate into a special region lacking companion cells (CCs), which is markedly different from the staggered pattern of SEs and CCs in healthy phloem (Xie et al., 2014), suggesting a process similar to (programmed) cell death in the infected tissues. However, it is not known which viral product is responsible for this cell death.

*Southern rice black‐streaked dwarf virus* (SRBSDV) (Zhou et al., 2008), also known as *Rice black‐streaked dwarf virus 2* (RBSDV‐2) (Zhang et al., 2008), is a newly recognized member of genus *Fijivirus* (Adams et al., 2016). The virus is a destructive pathogen that has been threatening the production of rice, maize, and other gramineous crop plants in East Asia in recent years (Lv et al., 2017b). Its virion is icosahedral but appears spherical in shape and consists of two concentric layers of capsid protein with an overall diameter of 75–85 nm, surrounding 10 linear double‐stranded (ds) RNA genome segments (Hoang et al., 2011). In the field, the virus is transmitted by the white‐backed planthopper (WBPH, *Sogatella furcifera*), a migratory pest, in a persistent‐propagative manner and the viral disease has thus rapidly expanded among rice‐growing areas of Vietnam, China, and Japan in recent years. In China alone, its outbreaks in 2010 affected more than 1,360,000 ha of rice‐growing areas, resulting in 30%–50% yield losses and even no harvest in some seriously infected rice paddies (Zhou et al., 2013). The viral 10 linear dsRNA genomic segments are named S1–S10 according to their migration from slow to fast in polyacrylamide gel electrophoresis (PAGE) (Wang et al., 2010). Genomic sequence analysis shows that S1–S4, S6, S8, and S10 are monocistronic, encoding proteins P1–P4, P6, P8, and P10, respectively, but S5, S7, and S9 each contain two open reading frames (ORFs). The proteins encoded by the upstream ORFs are called P5‐1, P7‐1, and P9‐1, whereas those encoded by the downstream ORFs are called P5‐2, P7‐2, and P9‐2. The entire genome of SRBSDV thus has at least 13 viral genes, of which six, P1‐P4, P8, and P10, encode structural proteins that are assembled into the virions (Lv et al., 2017b; Wang et al., 2010). Biochemical and immunological experiments indicate that P5‐1, P6, and P9‐1 are involved in the formation of viroplasms (Li et al., 2013, 2015), a type of discrete cytoplasmic inclusion body that is a factory for virus replication and assembly in infected host plants (Xie et al., 2017a) and vector planthoppers (Jia et al., 2012; Mao et al., 2013). The nonstructural protein P7‐1 is a viral component of the tubular structures that are typical in reoviral infections (Liu et al., 2011) and which are thought to play key roles in the intercellular movement of virions within insects (Jia et al., 2014). The other proteins of SRBSDV also seem to be similar to their counterparts in other fijiviruses in size and amino acid sequences, and may be involved in virus–host or virus–vector interactions; however, their functions remain unknown.

In host plants, SRBSDV induces some typical symptoms, including dwarfing and swellings or tumours, which are often found along the leaf sheath or on the underside of the leaf blade, and along the veins on stem of rice (Wu et al., 2013) and maize (Hoang et al., 2011). These swellings or tumours are derived from the hyperplasia of phloem tissues but are obviously different from healthy phloem tissues in cell types and frequency of intercellular gateways, which may provide a better microenvironment for SRBSDV multiplication and movement (Xie et al., 2014). In the SRBSDV‐induced tumours, SE hyperplasia and the special region composed exclusively of SE are also observed. As the process of SE differentiation resembles an arrested cell death (Furuta et al., 2014; Geldner, 2014), we suggested that SRBSDV might encode a protein pronouncedly affecting cell viability. To test this hypothesis, we have now investigated the ability of each SRBSDV‐encoded protein to induce cell death in plant cells and/or in the bacterium *Escherichia coli*.

## 2 RESULTS

### 2.1 Screening of SRBSDV‐encoded proteins that affect plant cell viability

**FIGURE 1:**
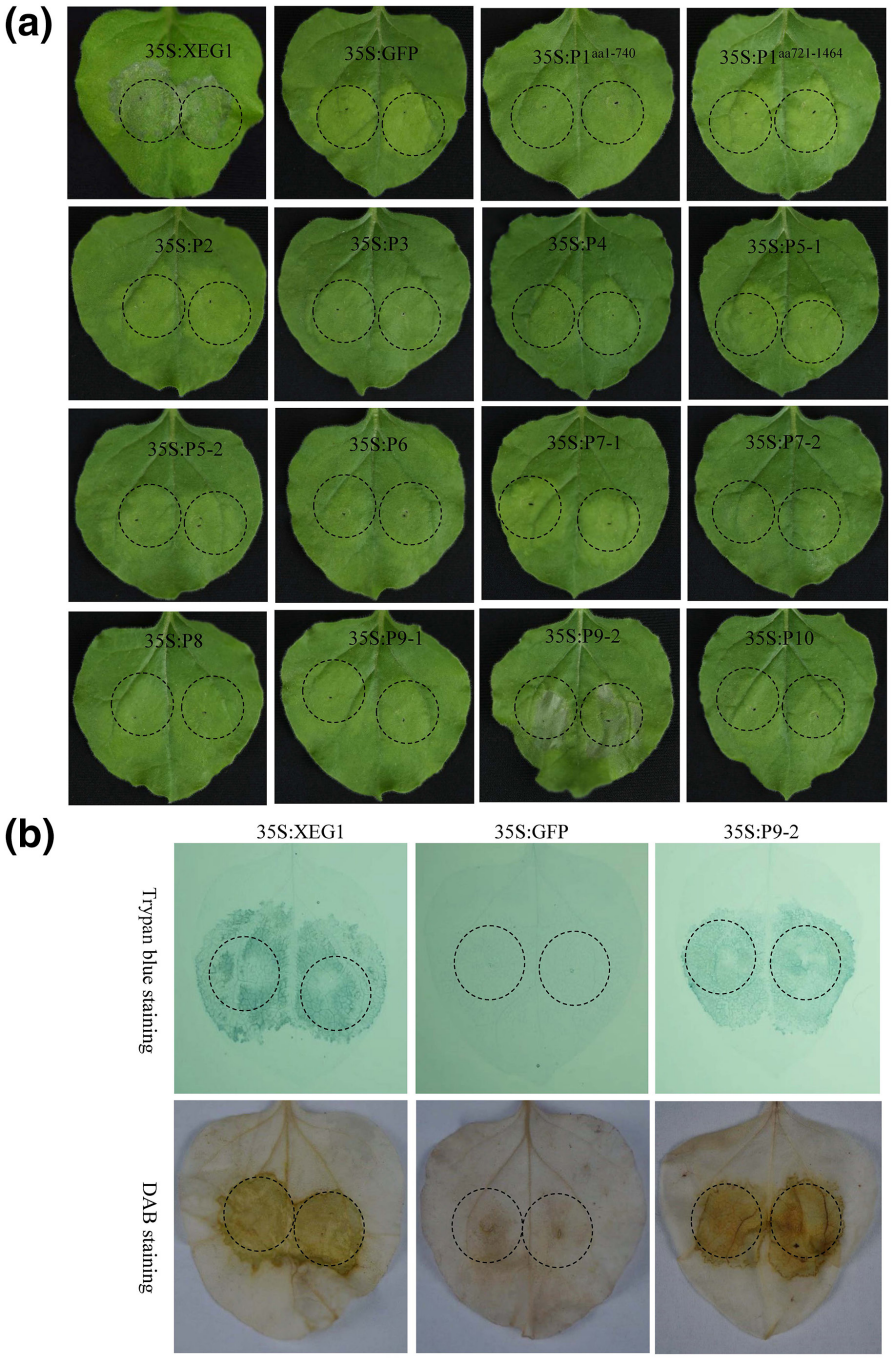
Identification of SRBSDV proteins involved in cell death in leaves of *Nicotiana benthamiana* by *Agrobacterium tumefaciens*‐mediated transient expression (a) and histochemical staining (b; trypan blue and 3,3′‐diaminobenzidine [DAB]), in which the transient expression of XEG1 and GFP were used as positive and negative control, respectively. Photographs taken 3 days after agroinfiltration.

To determine whether protein(s) encoded by SRBSDV have the ability to induce cell death, the 13 SRBSDV‐encoded proteins were individually expressed by *Agrobacterium tumefaciens*‐mediated transient expression, a procedure that has been widely used to detect the cytopathogenic effects of viral (Qian et al., 2016) or nonviral effector proteins (Franco‐Orozco et al., 2017; Yu et al., 2012). Briefly, each SRBSDV protein was expressed under the control of double cauliflower mosaic virus (CaMV) 35S promoters in *Nicotiana benthamiana* leaves through agroinfiltration. The jellyfish green fluorescent protein (GFP) was used as a negative control, with XEG1, a *Phytophthora sojae* effector protein that causes cell death, used as a positive control in plants (Ma et al., 2015). As expected, necrosis was observed 3 days after agroinfiltration in leaf patches expressing XEG1 but not in patches expressing GFP (Figure 1a). Interestingly, SRBSDV P9‐2, a nonstructural protein of unknown function, was also observed to induce necrosis similar to, although less pronounced than, that caused by XEG1 (Figure 1a). All other SRBSDV proteins behaved like GFP in agroinfiltrated leaf patches (Figure 1a). Trypan blue staining, an assay for the visualization of cell death (Cooksey, 2014; Keogh et al., 1980), further confirmed the occurrence of cell death in the leaf patches agroinfiltrated with the 35S:P9‐2 construct (Figure 1b) as in those expressing XEG1, clearly indicating that P9‐2 could induce cell death in plant cells.

The burst of reactive oxygen species (ROS) is thought to be a hallmark of cell death (Breusegem & Dat, 2006; Fulda, 2016). Consistent with this, leaf patches expressing XEG1 or P9‐2, but not those expressing GFP or other SRBSDV proteins, could be readily stained by 3,3′‐diaminobenzidine (DAB), a dye that has been widely used to detect H_2_O_2_ (Figure 1b) (Thordal‐Christensen et al., 1997), demonstrating that P9‐2 also induced cell death by a burst of ROS.

### 2.2 P9‐2 induces systemic symptoms with necrosis when expressed by a heterologous virus

**FIGURE 2:**
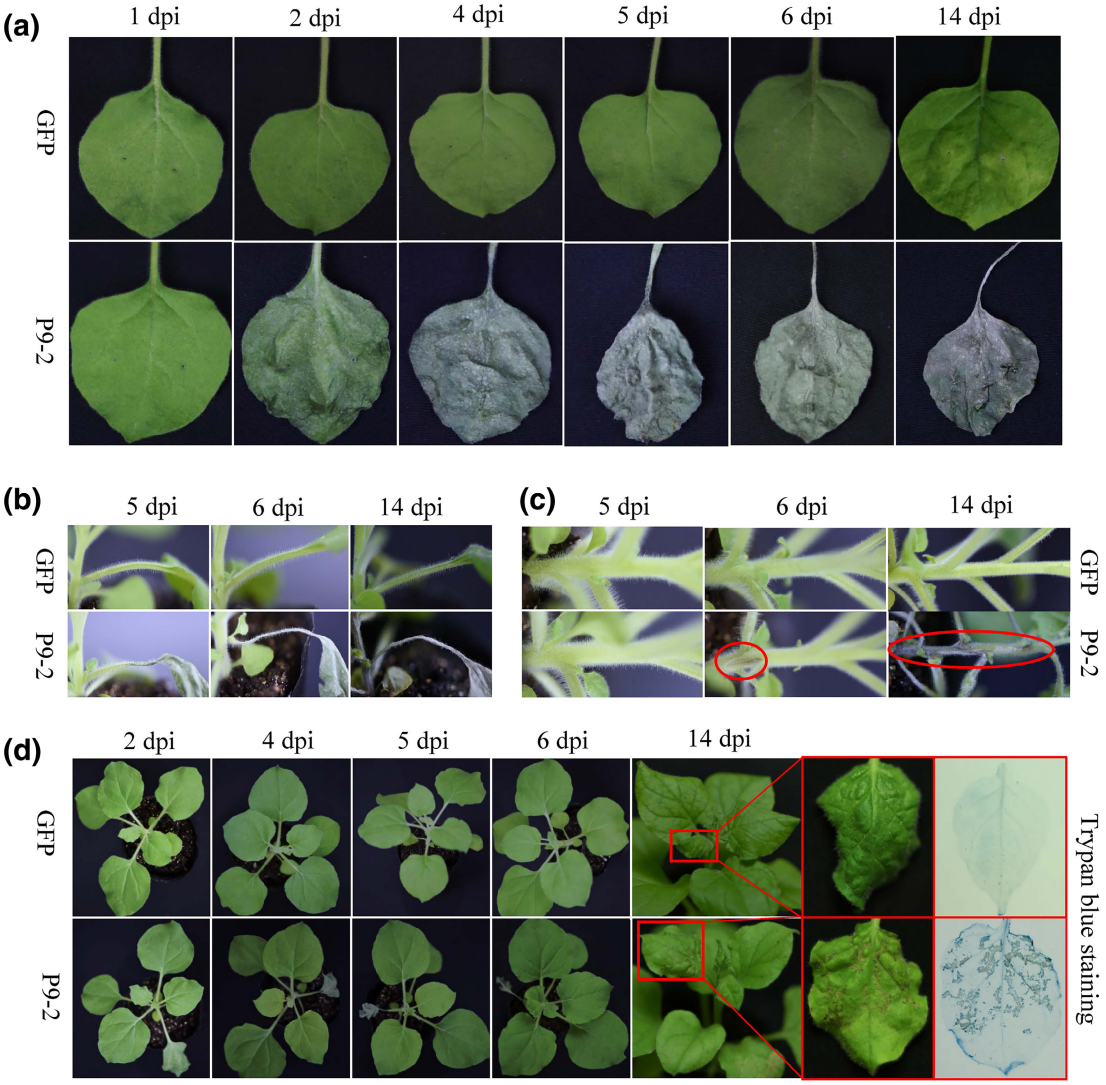
Symptoms on the inoculated leaves (a), petioles (b), stems (c), and upper leaves (d) of *Nicotiana benthamiana* plants inoculated with TRV expressiing the SRBSDV protein P9‐2. TRV expressing GFP was used as a negative control. dpi, days postinoculation.

To investigate whether P9‐2 induces plant cell death when expressed by a different virus, the P9‐2 ORF was cloned into the tobacco rattle virus (TRV) vector pTRV2 (Liu et al., 2002). *Agrobacterium* cultures harbouring this recombinant plasmid were infiltrated into *N. benthamiana* leaves together with those harbouring pTRV1. Like the transient assay described above, necrotic spots developed in infiltrated leaf patches (Figure 2a). Unlike the transient assay, however, the necrotic spots enlarged progressively, culminating in death of the entire leaf (Figure 2a).

Systemic infection was observed in almost all of the inoculated plants. In these plants, necrosis was observed along the petiole of the inoculated leaf at 4–5 days postinoculation (dpi) and along the stem region adjacent to the petiole at 6 dpi (Figure 2b,c). At 14 dpi, the entire stem became grey and brown (Figure 2c). By this time, lesions were observable on upper uninoculated leaves (Figure 2d). Cell death in and around these lesions was confirmed by trypan blue staining (Figure 2d). By comparison, TRV‐GFP induced very mild symptoms in upper leaves but without any necrosis on either the inoculated or systemic leaves (Figure 2a–d). TRV has been widely used as a tool to silence endogenous genes (Liu et al., 2002). To rule out the possibility that the cell death induced by TRV‐P9‐2 might be caused by silencing of an unknown plant gene, TRV carrying the P9‐2 ORF but without a start codon (TRV‐ΔP9‐2) was constructed. *N. benthamiana* inoculated with this construct showed no difference to those inoculated with TRV‐GFP in both inoculated and systemic leaves (Figure S1). Thus, even if the small interfering RNAs derived from the P9‐2 ORF targeted some endogenous genes, this did not lead to cell death, supporting the view that cell death is induced by P9‐2 at the level of protein rather than RNA.

### 2.3 P9‐2 induces cell death in *E. coli*

**FIGURE 3:**
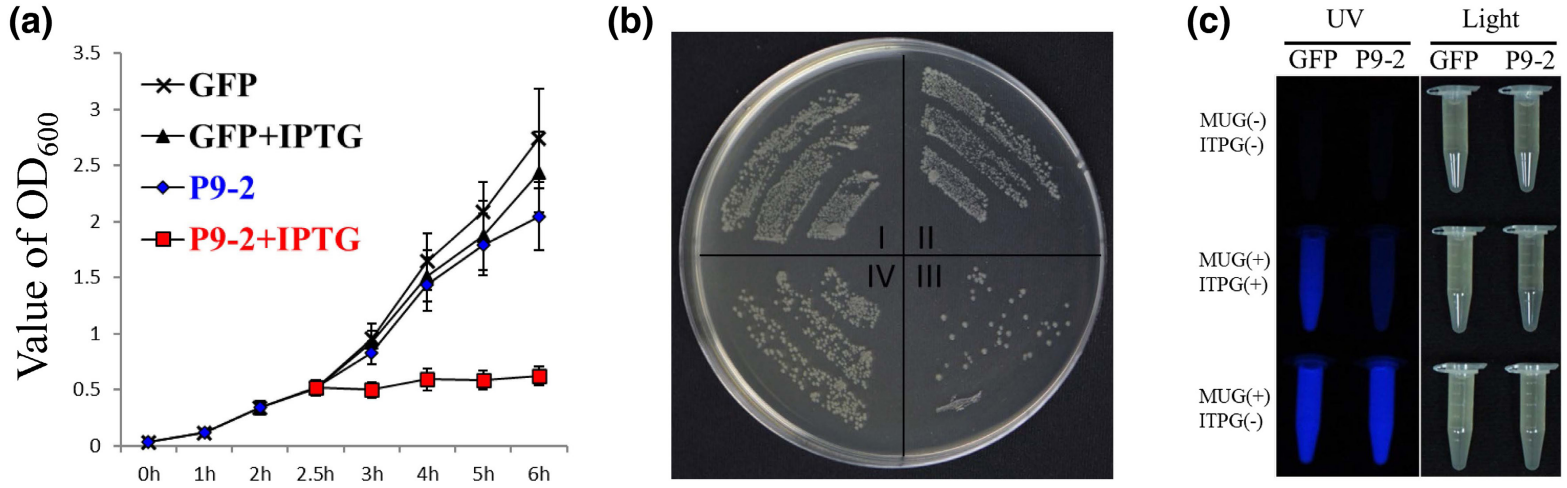
SRBSDV P9‐2 induced cell death in *Esherichia coli*. (a) The growth curves of *E. coli* BL21 (DE3) pLysS containing pET32a‐GFP or pET32a‐p9‐2 grown in Luria‐Bertani (LB) broth with or without isopropyl‐β‐D‐thiogalactoside (IPTG) induction. Cell growth was monitored by OD_600_. (b) *E. coli* BL21 (DE3) pLysS containing pET32a‐GFP (I and II) or pET32a‐p9‐2 (III and IV) were growth on an LB plate for 16 h with (II and III) or without IPTG (I and IV). (c) 4‐methylumbelliferyl‐β‐D‐glucuronic acid (MUG) was added to overnight cell cultures of the *E. coli* BL21 (DE3) pLysS containing pET32a‐p9‐2/pET32a‐GFP, alone or together with IPTG. At 3 h after the addition, the cultures of *E. coli* BL21 (DE3) pLysS growth were monitored under UV light. Each experiment was performed at least three times.

In attempts to prepare an antiserum against P9‐2, we failed to express the protein in prokaryotic expression systems. This prompted us to test the idea that P9‐2 might also induce cell death in prokaryotic cells. The P9‐2 ORF was therefore subcloned into the prokaryotic expression vector pET32a and the resulting plasmid, pET32a‐P9‐2, was transformed into *E. coli* BL21 (DE3) pLysS. pET32a‐GFP was obtained similarly and used as a negative control. In the absence of isopropyl‐β‐D‐thiogalactoside (IPTG), an inducer for prokaryotic expression, the growth of the *E. coli* carrying pET32a‐P9‐2 was comparable to, although slightly slower than, those carrying pET32a‐GFP (Figure 3a). IPTG had negligible effects on the *E. coli* carrying pET32a‐GFP. However, it seriously inhibited the growth of *E. coli* carrying pET32a‐P9‐2 (Figure 3a). Aliquots of each culture were pipetted out 3.5 h after IPTG addition, plated on solid Luria‐Bertani (LB) medium after a 1000‐fold dilution and allowed to grow overnight. As shown in Figure 3b, numerous colonies were found on the medium plated with the *E. coli* carrying pET32a‐GFP, irrespective of IPTG addition, and also from the uninduced *E. coli* carrying pET32a‐P9‐2. However, only a small number of colonies were observed on the medium plated with the IPTG‐induced *E. coli* carrying pET32a‐P9‐2.

As an independent approach, 4‐methylumbelliferyl‐β‐D‐glucuronic acid (MUG) was added to overnight cell cultures of the *E. coli* carrying pET32a‐P9‐2/pET32a‐GFP, alone or together with IPTG. Three hours after the addition, the *E. coli* was observed under UV light. Because living *E. coli* can hydrolyse MUG to 4‐methylumbelliferone (4‐MU), which emits blue light under UV light excitation, the intensity of the blue fluorescence was an indicator of the amounts of living *E. coli* cells. As shown in Figure 3c, IPTG had no visible effect on the fluorescence intensity of *E. coli* carrying pET32a‐GFP, but it greatly reduced the fluorescence intensity of the *E. coli* carrying pET32a‐P9‐2, showing that the expression of P9‐2 also could destroy the viability of prokaryotic cells.

### 2.4 Plasma membrane localization of P9‐2 is required for cell death induction

**FIGURE 4:**
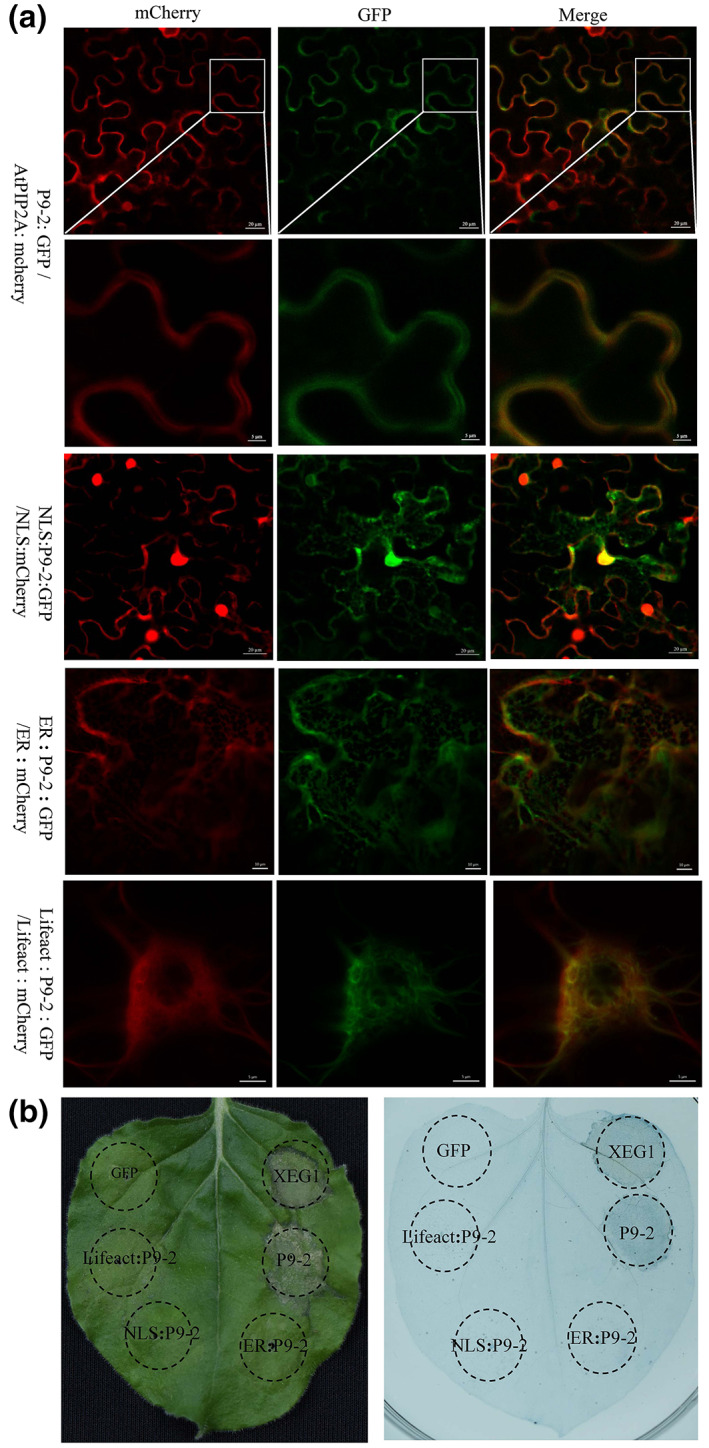
Plasma membrane localization of P9‐2 is required for cell death induction. (a) P9‐2 was colocalized with AtpPIP2A, a marker labelling the plasma membrane in plant cells. (b) Modification of P9‐2 subcellular localization abolished its ability to induce cell death in plant cells. ER, endoplasmic reticulum signal peptide/retention signal (HDEL); Lifeact, marker to visualize F‐actin; NLS, nuclear localization signal from SV40. GFP and XEG1, negative and positive controls, respectively.

Subcellular localization of a protein may provide a clue to its functions and we therefore predicted the subcellular localization of P9‐2 using two independent web‐based programs that make predictions based on the protein amino acid sequences: PSORT v. 6.4, (https://psort.hgc.jp/) and YLoc‐HighRes (https://abi‐services.informatik.uni‐tuebingen.de/yloc/webloc.cgi). Both packages indicated that P9‐2 might most probably (60%–80%) be targeted to plasma membranes. To validate its subcellular localization, binary vectors expressing P9‐2 fused at its N‐ or C‐terminus with the GFP and *Arabidopsis* plasma membrane intrinsic protein 2A (AtPIP2A), a marker labelling plasma membrane in plant cells (Nelson et al., 2007), fused with m‐Cherry, were constructed and coinfiltrated into *N. benthamiana* epidermal cells using the *Agrobacterium*‐mediated method. To decrease the effect of cell death on the subcellular localization, the fluorescence was monitored by confocal microscopy 30 h after infiltration, an early stage of cell death. In the coinfiltrated cells, the fluorescence from fused P9‐2/GFP was mainly distributed on plasma membranes and most of the GFP fluorescence was colocalized with that from fused AtPIP2A/mCherry (Figure 4a). In a subcellular fractionation assay, P9‐2 was mainly detected in the plasma membrane compartment (Figure S2), further supporting its subcellular localization on the plasma membrane. Interestingly, when its N‐terminus was fused with a sequence for the nuclear localization signal (NLS) from SV40 (Kalderon et al., 1984), the modified P9‐2 protein was transported into the nucleus (Figure 4a) but did not induce cell death at all (Figure 4b), thus behaving like GFP. Similar results (Figure 4a,b) were observed when P9‐2 was artificially relocalized into the endoplasmic reticulum (ER) lumen or bound onto the F‐actin cytoskeleton by attaching an ER signal peptide/retention signal (HDEL) (Gomord et al., 1997; Nelson et al., 2007) or Lifeact, a 17 amino acid peptide used as a versatile marker to visualize F‐actin (Lv et al., 2017c; Riedl et al., 2008). Taken together, these results indicate that the plasma membrane localization of P9‐2 protein could be required for its ability to induce cell death.

### 2.5 Transmembrane helices of P9‐2 were crucial for cell death induction

**FIGURE 5:**
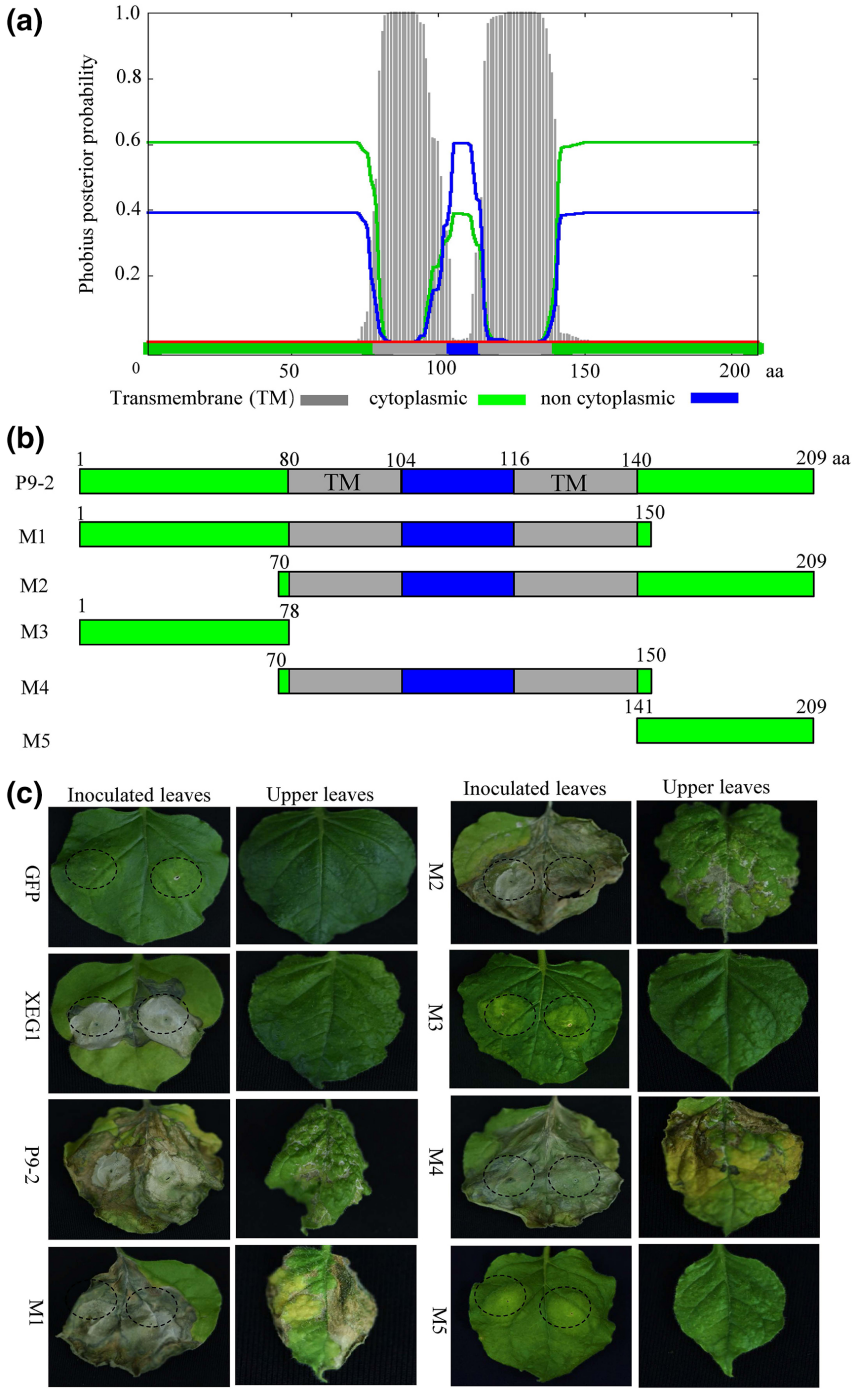
Transmembrane helices of P9‐2 were crucial for cell death induction. (a) Two transmembrane helices (TM) are in the central hydrophobic region of P9‐2. (b) Five truncated mutants of P9‐2. (c) Ability of mutants M1 to M5 to induce cell death on the inoculated and upper leaves of *Nicotiana benthamiana* by the TRV expression system. GFP and XEG1, negative and positive controls, respectively.

To further investigate its functional domains, two transmembrane helices of P9‐2 were identified spanning amino acid residues 80–104 and 116–140 using the transmembrane prediction servers, Phobius (https://phobius.sbc.su.se/) and TMHMM v. 2.0 (http://www.cbs.dtu.dk/services/TMHMM/) (Figure 5a). To determine the importance of the transmembrane helices in cell death induction, the full‐length (209 amino acids) P9‐2 of SRBSDV was truncated into a series of partially overlapping mutants (Figure 5b): M1 (amino acids 1–150), M2 (amino acids 70–209), M3 (amino acids 1–78), M4 (amino acids 70–150), and M5 (amino acids 141–209). Of these, M1, M2, and M4 retain the entire two transmembrane helices, which could maintain their plasma membrane localization (Figure S3), while M3 and M5 consist only of the N‐ or C‐ terminal parts and lack any complete transmembrane helix (Figure 5b). Each mutant was constructed and expressed by TRV as described above. As shown in Figure 5c, all three mutants harbouring the transmembrane helices (M1, M2, and M4) induced a large area of necrosis in inoculated leaves and readily detectable necrotic lesions in upper uninoculated leaves, similar to the cell death phenotype induced by the wild‐type P9‐2. In contrast, M3 and M5 induced almost no detectable necrosis in both the inoculated and upper leaves, thus behaving more like the negative control GFP. These data indicate that the two transmembrane helices of P9‐2 are necessary and sufficient for cell death induction in both local and systemic leaves.

The effects of M1–M5 expression on *E. coli* were also evaluated. The inducible expression of truncated mutants M1, M2, and M4 drastically decreased the viability of *E. coli* cells while expression of M3 and M5 had almost no influence on their growth (Figure S4). Thus, the central hydrophobic transmembrane region is also necessary and sufficient for the cell‐death induction activity of P9‐2 in *E. coli*.

**FIGURE 6:**
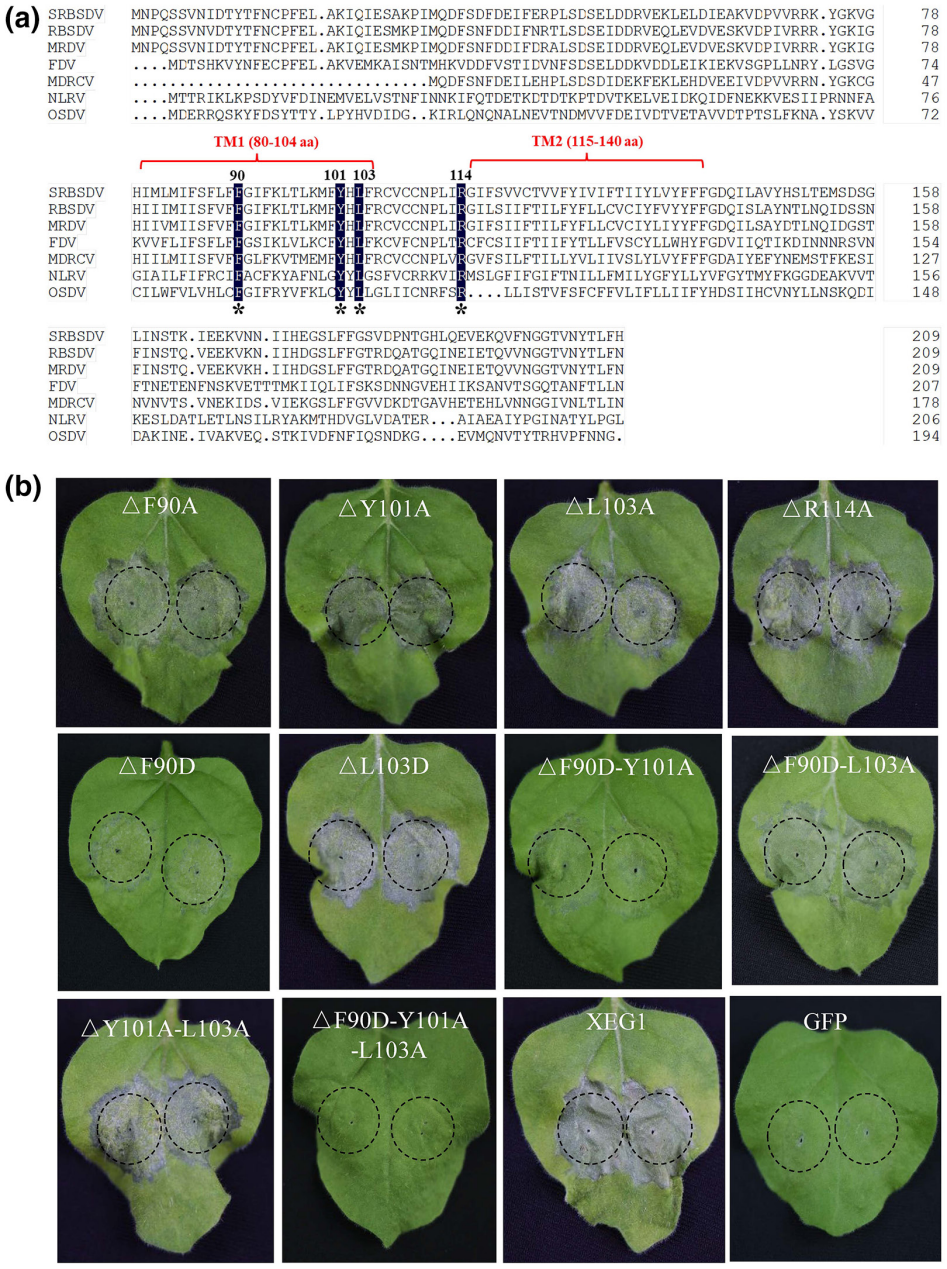
Site‐directed mutation assays of cell death in *Nicotiana benthamiana* plants. (a) Conserved amino acid residues among P9‐2 of fijiviruses in transmembrane (TM) domains. (b) Ability of site‐directed mutants (Δ) to induce cell death in *N. benthamiana* plants. GFP and XEG1, negative and positive controls, respectively.

A similar central hydrophobic transmembrane region (Figure S5) is found in the P9‐2 of all other known fijiviruses, including *Fiji disease virus* (FDV) (Soo et al., 1998), *Rice black‐streaked dwarf virus* (RBSDV) (Isogai et al., 1998a), *Mal de Río Cuarto virus* (MRCV) (Guzmán et al., 2007; Maroniche et al., 2012), *Maize rough dwarf virus* (MRDV) (Lv et al., 2016), *Oat sterile dwarf virus* (OSDV) (Isogai et al., 1998b), and *Nilaparvata lugens reovirus* (NLRV) (Nakashima et al., 1996). An alignment of the region revealed four conserved sites, corresponding to a phenylalanine (F), a tyrosine (Y), a leucine (L), and an arginine (R) residue at amino acid positions 90, 101, 103, and 114 of SRBSDV P9‐2, respectively (Figure 6a). To test the importance of these sites in cell death induction, four SRBSDV P9‐2 mutants, each with one conserved residue substituted with alanine (A), were created and designated as P9‐2‐F90A, ‐Y101A, ‐L103A, and ‐R114A, respectively. At 48 h after inoculation, all the mutants appeared to induce cell death in infiltrated leaf patches (Figure 6b), although the mutant P9‐2‐F90A induced less cell death than the other three, which were comparable to the wild‐type P9‐2 (Figure 6b). These results suggested that F90 might play more important role in cell death induction. To validate this idea, the mutants P9‐2‐F90D and P9‐2‐L103D, in which F90 and L103 were substituted with aspartic acid (D), a polar amino acid residue with negative charge, were constructed and tested as above. The mutant P9‐2‐F90D appeared to induce much less cell death than any other single site‐directed mutant while the effects of P9‐2‐L103D were similar to those of P9‐2‐L103A and intact P9‐2, supporting the importance of F90. Because three of the four conserved sites were located within the first transmembrane helix, we hypothesized that the first transmembrane helix may be more important for cell death induction and that the conserved amino acids may have synergistic effects so that a single amino acid mutation might not be enough to abolish its function. Based on this idea, three double mutants of SRBSDV P9‐2 were created. All the three mutants were each expressed with the TRV vector (Figure S6) and they still appeared to induce cell death, although to varying degrees (Figure 6b). Among above mutants, the double mutant F90D‐Y101A was weakest in cell death induction while the mutant Y101A‐L103A still retained a much stronger ability to induce cell death than another double mutant F90D‐L103A (Figure 6b). By contrast, the triple mutant P9‐2‐F90D‐Y101A‐L103A, which has its Y101 and L103 substituted with A and F90 substituted with aspartate (D), did not induce any cell death in plant cells, just like TRV‐GFP (Figure 6b). Although further studies are needed to explain these observations, altogether they support the notion that the first transmembrane helix within the central hydrophobic region could be crucial for cell death induction and that the conserved F90, Y101, and L103 amino acid residues could play synergistic roles in maintaining the ability of P9‐2 to induce cell death.

### 2.6 P9‐2 activity is conserved in fijiviruses

**FIGURE 7:**
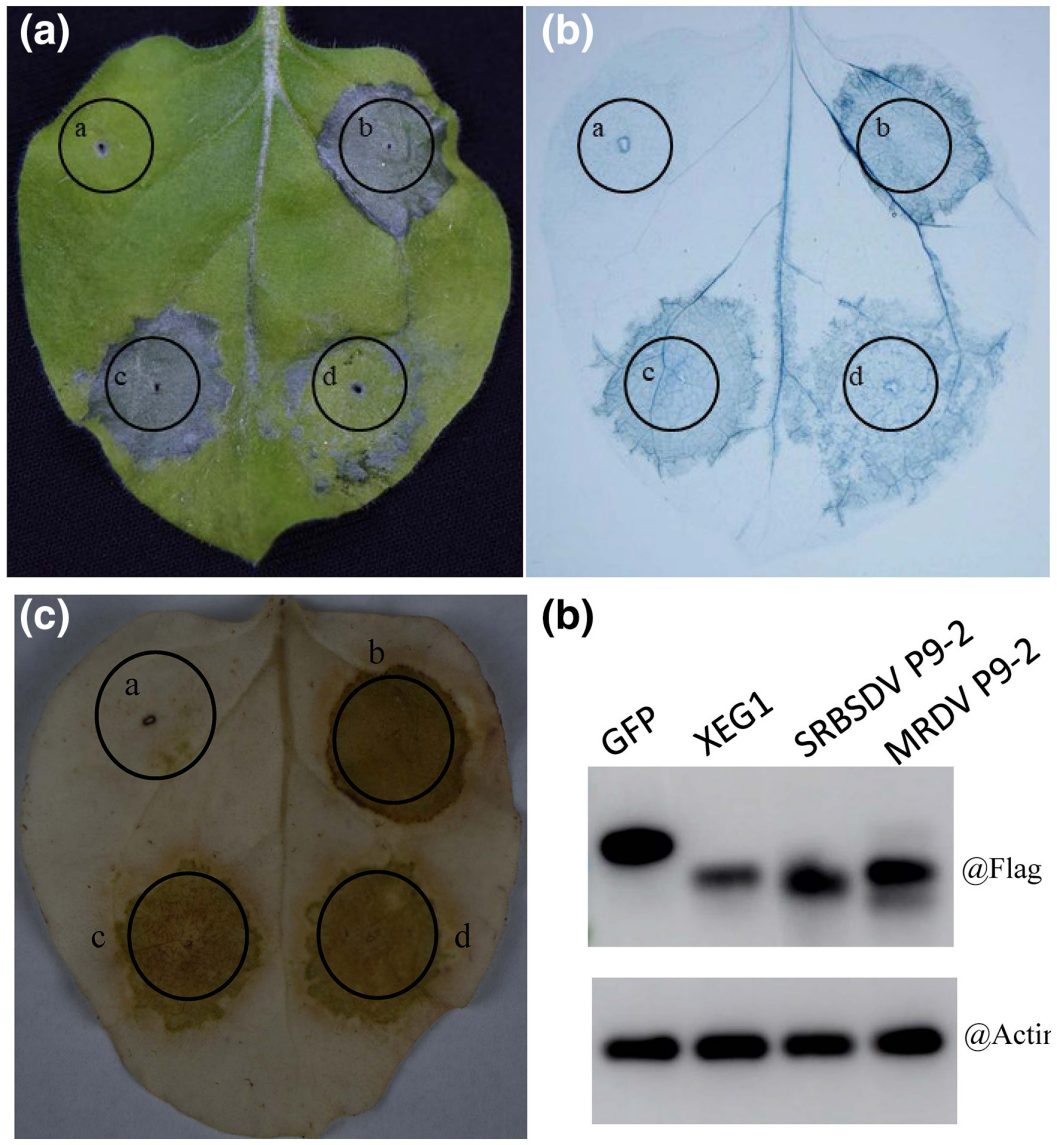
Transient expression of P9‐2 protein from *Maize rough dwarf fijivirus* (MRDV) in leaves of *Nicotiana benthamiana*. (a) Cell death symptoms on the inoculated leaf of *N. benthamiana*. (b) Histochemical staining with trypan blue. (c) 3,3′‐diaminobenzidine (DAB) staining. (d) Detection of MRDV P9‐2 protein in leaves of *N. benthamiana* by western blotting. In the transient expression assays, patches (a–d) were agroinfiltrated with constructs GFP, XEG1, SRBSDV P9‐2, or MRDV P9‐2, respectively, in which XEG1 and GFP were used as positive and negative controls.

SRBSDV P9‐2 and its homologues are conserved among fijiviruses, sharing 21%–73% amino acid identity (Xie et al., 2017b; Xue et al., 2014). However, these proteins do not exhibit any significant sequence similarity to other reoviral (or indeed any other viral) protein, suggesting that P9‐2 may be unique to fijiviruses. Further multiple alignment suggested that a central hydrophobic region was always present in fijiviral P9‐2 homologues and two transmembrane helices could be predicted using the transmembrane topology prediction servers, Phobius (https://phobius.sbc.su.se/) and TMHMM v. 2.0 (http://www.cbs.dtu.dk/services/TMHMM/) (Figure S5). The similarity suggests that the ability of P9‐2 homologues to induce cell death may be conserved among fijiviruses. To confirm this speculation, we also cloned the full‐length coding region of P9‐2 from *Maize rough dwarf virus* (MRDV), another member of the genus *Fijivirus* (Lv et al., 2016), and tested its cytopathogenic effects in a similar way. As expected, MRDV P9‐2 also induced cell death either when expressed transiently under the control of the CaMV 35S promoter or systemically by TRV (Figure 7) and histochemical staining further confirmed that the cell death induced by MRDV P9‐2 was preceded by a burst of ROS, suggesting a similar process of cell death induction. The cell death induction activities of P9‐2 homologues from other fijiviruses have not yet been determined but two different research groups have reported failure to express the full‐length P9‐2 of FDV (Soo et al., 1998) or RBSDV (Isogai et al., 1998a). In our previous studies to prepare antisera, we also failed to express the full‐length P9‐2 protein of RBSDV and SRBSDV due to poor cell survival rate. However, in these three independent studies, deletion of the central hydrophobic domain resulted in successful expression of the proteins from different fijiviruses, also suggesting that the central hydrophobic domains play an important role in the cell death induction. Thus, the cell‐death induction activity and the mechanisms underlying this activity may be universal for P9‐2 among all fijiviruses.

## 3 DISCUSSION

Our previous studies suggest that a product of SRBSDV may induce the SE differentiation by cell death‐like or a near‐death process, but an experimental system allowing us to identify the effect of a viral protein on SE differentiation is unavailable so far. However, because SE differentiation resembles a specific form of cell death (Furuta et al., 2014), the results from this study are at the least suggestive. Overexpression of P9‐2 induced cell death in both local and systemic leaves and in plants infiltrated with the full‐length P9‐2 and its mutants M1, M2, and M4, cell death was also readily observed in petioles and stems along the veins, indicating the induction of cell death in plant vascular tissues. Thus P9‐2 appears to be the best candidate for a viral protein that triggers the distinctive cytopathological effects.

Many proteins encoded by viral (TuMV P3, Kim et al., 2010; begomovirus V2, Mubin et al., 2010; MCMV P31, Jiao et al., 2021) or nonviral pathogens cause cell death in plants (*Xanthomonas* AvrBsT, Han & Hwang, 2017; CRP, Nie et al., 2019; *Magnaporthe oryzae*, Guo et al., 2019; *Botrytis cinerea* transglycosylase, Bi et al., 2021). Generally, one of the following two mechanisms is involved. First, the protein can be recognized by a resistance ( R) protein from the plant, either directly or indirectly. The R protein then triggers a signal transduction cascade, leading to rapid cell death (Coll et al., 2011; de Ronde et al., 2014; Sun et al., 2020). This type of cell death, commonly known as a hypersensitive reaction, is a defence mechanism used by plants to combat infections (Kombrink & Schmelzer, 2001; Park, 2005). Second, the protein may activate or hijack a prodeath signal cascade. In this case, the cell death induced by the protein can be blocked by silencing one or several host genes (Yu et al., 2012; Zhang et al., 2020). In *N. benthamiana* plants, P9‐2 appeared to activate the expression of marker genes for programmed cell death (PCD) (Figure S7), supporting that the viral protein could induce a process resembling PCD in plants. Because *N. benthamiana* is not a host of SRBSDV or any related fijiviruses, it seems unlikely that this plant has an R protein to specifically recognize P9‐2. Therefore, we prefer the second possibility for the cell death induction of P9‐2.

Our results showed that P9‐2 also induces cell death in *E. coli*, a model prokaryotic cell. The hydrophobic domain necessary for cell death induction in plants is also important for cell death induction in *E. coli*, suggesting that the same mechanism is used by P9‐2 to induce cell death in the two very different systems. This seems to be a very surprising finding. Many studies have shown that prokaryotic microbes can undergo PCD in a manner like higher eukaryotes. Some mammalian prodeath proteins such as Bax (Kawai‐Yamada et al., 2001; Lacomme & Cruz, 1999), FADD‐DED (Fas‐associated death domain‐death effector domain; Yan et al., 2013), and HAP (homologue of apoptosis/saibousi Yutsudo [ASY] protein) induce cell death in bacteria (Gan et al., 2004). The cell death is preceded by physiological changes like those found in mammalian cells undergoing PCD, pointing to a conserved mechanism. Thus, it is possible that P9‐2 may activate a prodeath pathway that is conserved among prokaryotes and eukaryotes. In this regard, it is worth noting that HAP contains two transmembrane domains like P9‐2, although the role of the transmembrane domains in the action of HAP has yet to be determined (Gan et al., 2004).

All fijiviruses encode P9‐2 or a homologue as the downstream ORF within corresponding genomic positions (FDV, Soo et al., 1998; RBSDV, Isogai et al., 1998a; MRCV, Guzmán et al., 2007; MRDV, Xie et al., 2017b; SRBSDV, Xue et al., 2014). Our previous studies showed that no subgenomic RNAs for downstream ORFs can be detected in fijivirus‐infected plants and that there are no known internal ribosome entry sites to express these 3′‐proximal ORFs of bicistronic dsRNAs (Lv et al., 2012; Yang et al., 2014). The only plausible mechanism by which these downstream ORFs can be expressed is restarting or leaky scanning (Li et al., 2011), which is supported by the very low levels of P5‐2 (Yang et al., 2014) and P7‐2 (Lv et al., 2012) when compared with those of their upstream ORFs in fijivirus‐infected plants. It seems reasonable that the accumulation levels of P9‐2 in SRBSDV‐infected plants would be very low. By contrast, both the *Agrobacterium*‐mediated transient assay and the TRV‐mediated heterologous expression used in this study produced high levels of P9‐2. From this point of view, the cell‐death induction of P9‐2 demonstrated here may not be comparable to natural viral infection and might dramatically enhance the cytopathological effects of the protein. This suggests that future studies into the effects of P9‐2 on SE differentiation might best be conducted using lower expression levels. However, the findings of this study may still have biological significance for at least the following two putative reasons: (i) P9‐2 may induce the death of certain cell type (i.e., companion cell) at low concentrations, as predicted from the idea that it may function through triggering a signal cascade, which might lead to lack of companion cells within SRBSDV‐induced tumours (Xie et al., 2014). (ii) P9‐2 may induce a very mild form of cell death or near‐death differentiation when expressed at low concentrations, which might regulate SE specification and differentiation from the division of phloem‐parenchyma cells, leading to SE hyperplasia and the de novo formation of an SE‐SE specific region within SRBSDV‐induced tumours (Xie et al., 2014). Although the information on the ontogeny of SRBSDV‐induced SE‐SE specific region and the role of P9‐2 in SE differentiation remains very limited, this is therefore the first step to dissect a viral factor that may be involved in SE differentiation, which may link SE differentiation to a mild form of cell death.

## 4 EXPERIMENTAL PROCEDURES

### 4.1 Plant materials

All *N. benthamiana* plants were grown in a growth chamber at 25°C with 16 h light/8 h dark and 70% relative humidity as previously described. Infected leaves were collected from diseased rice plants in Zhejiang Province and stored at −80°C until use.

### 4.2 Plasmid construction

A pCAMBIA L1300‐derived binary vector (Figure S8) was used for transient expression. The ORFs specifying GFP, XEG1 or SRBSDV proteins were each amplified from GFP‐ or XEG1‐containing plasmids or SRBSDV genomic cDNAs by PCR with primer pairs (Table S1). The PCR products were recombined into the pDONR201 Gateway vector with BP clonase (Invitrogen) and sequenced, before inserting into the binary vector with LR clonase (Invitrogen).

The TRV‐based vector (Figure S9) was used to express GFP, XEG1, P9‐2 or P9‐2 mutants in *N. benthamiana*. To do this, each fragment of interest was amplified by conventional or overlapping PCR with primer pairs (Table S2) and the PCR product was inserted into the *Cla*I/*Sal*I site of the vector pTRV2 using a ClonExpress II One Step Cloning Kit (Vazyme).

pET32a (Novagen) was used to express GFP, P9‐2 or P9‐2 mutants in *E*. *coli*. To do this, each fragment of interest was amplified by PCR with primer pairs (Table S3) and the PCR product was inserted into the *Eco*RI/*Xho*I site of the vector pET32a.

For subcellular localization studies, the expression cassettes of P9‐2/GFP and AtPIP2A/mCherry fusions were inserted in a modified binary vector from pCAMBIA1300 (Figure S8). To change the subcellular localization, the N‐ or C‐ends of P9‐2 were fused with the nuclear localization signal (NLS), endoplasmic reticulum (ER) signal peptide/retention signal (HDEL), or Lifeact sequences. The primer pairs for plasmid construction are listed in Table S4 and their schematic diagrams are shown in Figure S8.

### 4.3 Agroinfiltration

Each recombinant binary construct described above was transformed to *Agrobacterium tumefaciens* GV3101 by electroporation (Bio‐Rad Gene Pulser, 0.2 cm cuvettes, 25 μF, >2.5 kV). Positive transformants were verified by PCR and cultured in yeast extract peptone (YEP) liquid medium supplemented with the appropriate antibiotics. After growth at 28°C with shaking, the bacterial cells were harvested and resuspended in induction medium (10 mM 2‐(N‐morpholino)ethanesulfonic acid [MES], pH 5.7, 10 mM MgCl_2_, 200 mM acetosyringone). Bacterial cells harbouring recombinant binary plasmid were adjusted to a final OD_600_ of 1.0, while those harbouring recombinant pTRV2 were adjusted to a final OD_600_ of 0.8 before mixing with a bacterial culture (OD_600_ = 0.8) harbouring pTRV1 at a ratio of 1:1. The bacterial suspension was incubated at room temperature for 2–4 h and then infiltrated into the underside of 30‐ to 40‐day‐old *N. benthamiana* leaves using a 1 ml syringe. After infiltration, the plants were kept in a greenhouse at 25°C under a 16 h light/8 h dark cycle and their phenotypes were surveyed daily.

### 4.4 DAB and trypan blue staining

DAB staining was performed according to a procedure described by Thordal‐Christensen et al. (1997). Briefly, leaves were excised from *N. benthamiana* using a sterilized razor blade at 72 h after agroinfiltration. After incubation for 8 h in a solution of 1 mg/ml DAB‐HCl (pH 3.8) at room temperature, the leaves were boiled in ethanol for 10 min and rinsed twice in double distilled water before photographing.

Trypan blue staining was performed according to a procedure described by Keogh et al. (1980) and Koch and Slusarenko (1990). Briefly, leaves were excised from *N. benthamiana* 72 h after agroinfiltration. After washing with double distilled water, the leaves were immersed and boiled in a trypan blue staining solution (10 ml lactic acid, 10 ml glycerol, 10 g phenol, 10 mg trypan blue, dissolved in 10 ml double distilled water) for 3 min, cooled at room temperature for 1 h, decoloured in 2.5 g/ml chloral hydrate for 48 h, and photographed.

### 4.5 Western blotting

Agroinfiltrated leaves were harvested and ground in liquid nitrogen. The powder (about 100 mg) was mixed with 100 μl of 2 × SDS loading buffer. After boiling for 5 min, the extracts were centrifuged at 12,000 × *g* for 5 min. The supernatant was loaded to and separated by 12.5% SDS‐PAGE. Separated proteins were transferred to a nitrocellulose membrane and were probed with a commercially available antibody to the FLAG epitope (TransGen). A goat antirabbit immunoglobulin G (IgG) conjugated with alkaline phosphatase (Sigma‐Aldrich) was used as the secondary antibody and the band was visualized by incubating the membranes in NBT‐BCIP solution following the protocol from the manufacturer.

### 4.6 Subcellular fractionation

An assay of cytoplasmic, nuclear, and membrane fractionation was performed using the Plant Nuclei and Cytoplasmic Protein Extraction Kit (BestBio) and Minute Plasma Membrane Protein Isolation kit (Invent) according to the manufacturer's protocols, respectively. The isolated proteins were subjected to SDS‐PAGE and immunoblotting as described above. The primary antibodies against H^+^ATPase (Agrisera), UDP‐glucose pyrophosphorylase (UGPase) (Agrisera), and histone H3 (Abclonal), were used as internal cellular compartment markers for plasma membrane, cytoplasm, and nucleus, respectively.

### 4.7 The effects of P9‐2 on *E. coli*

To obtain growth curves of *E. coli* expressing GFP, P9‐2 or P9‐2 mutants, each strain of *E. coli* was cultured in LB liquid medium supplemented with ampicillin at 37°C. An aliquot of the culture was inoculated to 10 ml of fresh LB and cultured to an OD_600_ of about 0.5. An aliquot of the second culture was inoculated to 40 ml of fresh LB. The liquid, with an OD_600_ of about 0.03, was cultured at 37°C with shaking. About 100 μl of the culture was pipetted out every 1 h for OD_600_ measurement. When the value of OD_600_ reached 0.5, IPTG was added to the culture at a final concentration of 0.4 μM. OD_600_ was measured every 0.5 h after IPTG addition.

To confirm cell death with MUG, a procedure described by Feng and Hartman (1982) was employed. Briefly, the *E. coli* BL21 (DE3) pLysS carrying each recombinant construct was cultured in LB liquid medium supplemented with ampicillin. An aliquot of the culture was inoculated to 10 ml of fresh LB and cultured to an OD_600_ of 0.5–0.8. The cells were harvested by a centrifuge at 3000 × *g* for 3 min and the pellet was resuspended with 2 ml of LB. The 2 ml of suspension was divided into two tubes. IPTG was added to both tubes at a final concentration of 0.4 μM, but MUG was added to only one of the two tubes at a final concentration of 100 μg/ml. The bacteria in each tube were cultured for 3 h before being observed under a long‐wave UV lamp.

### 4.8 Confocal microscopy

Fluorescence analysis was performed using a TCS SP5 confocal laser scanning microscope (Leica). GFP was excited at 488 nm and the emitted light captured at 500–550 nm, and mCherry was excited at 561 nm and emission light captured at 570–630 nm. For analysis of colocalization assays, multitracking was used to prevent emission cross‐talk between the channels. Images were captured digitally and handled using Leica TCS software. Postacquisition image processing was done with Photoshop v. 7.0 software (Adobe Systems Inc.).

## CONFLICT OF INTEREST

The authors declare no conflict of interest.

## Supporting information


**Figure S1** Expression of SRBSDV P9‐2 in leaves of *Nicotiana benthamiana* by the tobacco rattle virus (TRV) system. (a) Leaves inoculated with the TRV vector system harbouring XEG1, GFP, P9‐2, and ΔP9‐2 (a mutant without a start codon) (up) and their staining with trypan blue (down). (b) Detection of P9‐2:3 × FLAG expression in *N. benthamiana* at 48 h postinoculation by western blot with antibody against FLAG epitope. The antibody against SRBSDV P9‐2 was not yet available. To test the P9‐2 protein expressed from TRV vector, as an alternative, a 3 × FLAG tag was fused to the C terminus of P9‐2 (designed TRV‐P9‐2:3 × FLAG, which induced cell death like the TRV‐P9‐2 construct). As shown in Figure S1b, western blotting assay with the antibody against the FLAG epitope revealed a band with the expected size, which is marked with red box. M, marker for molecular weight of proteins

**Figure S2** Subcellular fractionation assay of SRBSDV P9‐2 expressed in *Nicotiana benthamiana* leaves. M, marker for molecular weight of proteins; PM, plasma membrane fraction; C, cytoplasm fraction; N, nuclear fraction

**Figure S3** P9‐2M4 mutant retaining the entire two transmembrane helices but deleting N‐ or C‐ terminal parts was colocalized with AtpPIP2A, a marker labelling plasma membrane in plant cells

**Figure S4** The ability of SRBSDV P9‐2 and its mutants to induce cell death in *Escherichia coli*

**Figure S5** A similar central hydrophobic transmembrane region was predicted in P9‐2 from all other six known fijiviruses, including *Fiji disease virus* (FDV), *Mal de Rio Cuarto virus* (MRCV), *Maize rough dwarf virus* (MRDV), *Rice black streaked dwarf virus* (RBSDV), *Oat sterile dwarf virus* (OSDV), and *Nilaparvata lugens reovirus* (NLRV)

**Figure S6** Detection of P9‐2 and its mutant proteins in *Escherichia coli* (a) and plant cells (b, c) by western blotting assays. (a) Inducible expression of SRBSDV P9‐2 in *E. coli*. (b) Expression of P9‐2 and its truncated proteins in leaves of *Nicotiana benthamiana* by the tobacco rattle virus system. (c) Expression of P9‐2 proteins with site‐directed mutations in *N. benthamiana* leaves. M, marker for molecular weight of proteins

**Figure S7** Relative expression level of 10 marker genes for programmed cell death (PCD) in *Nicotiana benthamiana* leaves agroinfiltrated with TRV‐GFP, ‐P9‐2 or ‐XEG1 plasmids. (a–d) Four vacuolar processing enzyme (VPE) genes, VPE1a, VPE1b, VPE2, and VPE3. (e) Zinnia endonuclease 1 (ZEN1). (f) Suppressor of G2 allele of Skp1 (SGT1). (g) Senescence‐associated gene 12 (SAG12). (h) Pathogenesis‐related 1a (PR1a). (i) Harpin‐induced gene 1 (HIN1). (j) Cys‐rich and transmembrane domain‐containing protein A‐like gene (CRTD)

**Figure S8** Schematic diagram of vector construction for transient expression or subcellular localization assays

**Figure S9** Schematic diagram of vector construction from tobacco rattle virus‐based expression system

**Table S1** Primer pairs used for the construction of transient expression in plant

**Table S2** Primer pairs for local and/or systemic expression from the tobacco rattle virus vector in this study

**Table S3** Primer pairs used for the construction of prokaryotic expression in *Escherichia coli*

**Table S4** Primer pairs used for vector construction in assays of subcellular localization

## Data Availability

The data supporting the findings of this study are available from the corresponding author upon reasonable request.
