# Supplementary figures and images for: A fijiviral nonstructural protein triggers cell death in plant and bacterial cells via its transmembrane domain
Source: Mol Plant Pathol. 2022 Oct 28;24(1):59–70. doi: 10.1111/mpp.13277 (PMC9742498; doi:10.1111/mpp.13277)

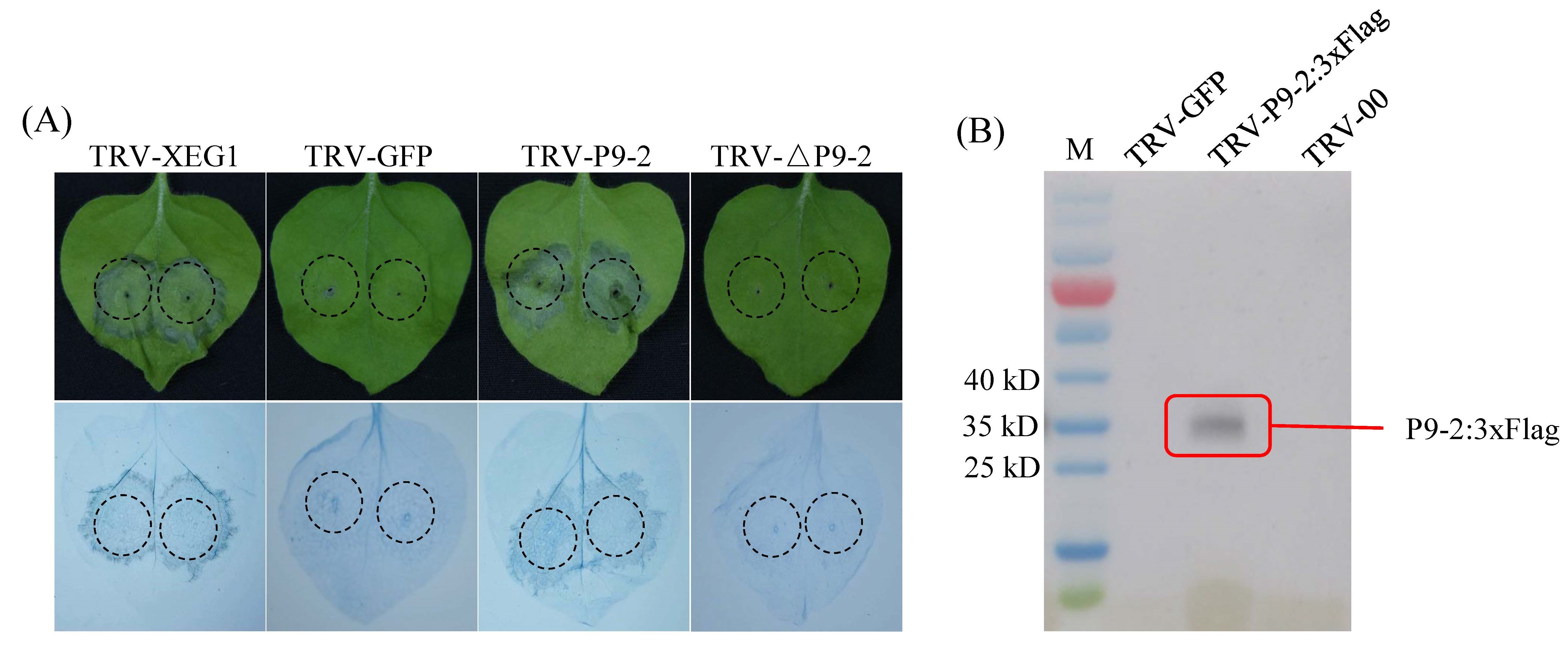

Supplement: Supplementary file 1 — Figure S1 Expression of SRBSDV P9‐2 in leaves of Nicotiana benthamiana by the tobacco rattle virus (TRV) system. (a) Leaves inoculated with the TRV vector system harbouring XEG1, GFP, P9‐2, and ΔP9‐2 (a mutant without a start codon) (up) and their staining with trypan blue (down). (b) Detection of P9‐2:3 × FLAG expression in N. benthamiana at 48 h postinoculation by western blot with antibody against FLAG epitope. The antibody against SRBSDV P9‐2 was not yet available. To test the P9‐2 protein expressed from TRV vector, as an alternative, a 3 × FLAG tag was fused to the C terminus of P9‐2 (designed TRV‐P9‐2:3 × FLAG, which induced cell death like the TRV‐P9‐2 construct). As shown in Figure S1b, western blotting assay with the antibody against the FLAG epitope revealed a band with the expected size, which is marked with red box. M, marker for molecular weight of proteins [file MPP-24-59-s012.jpg]

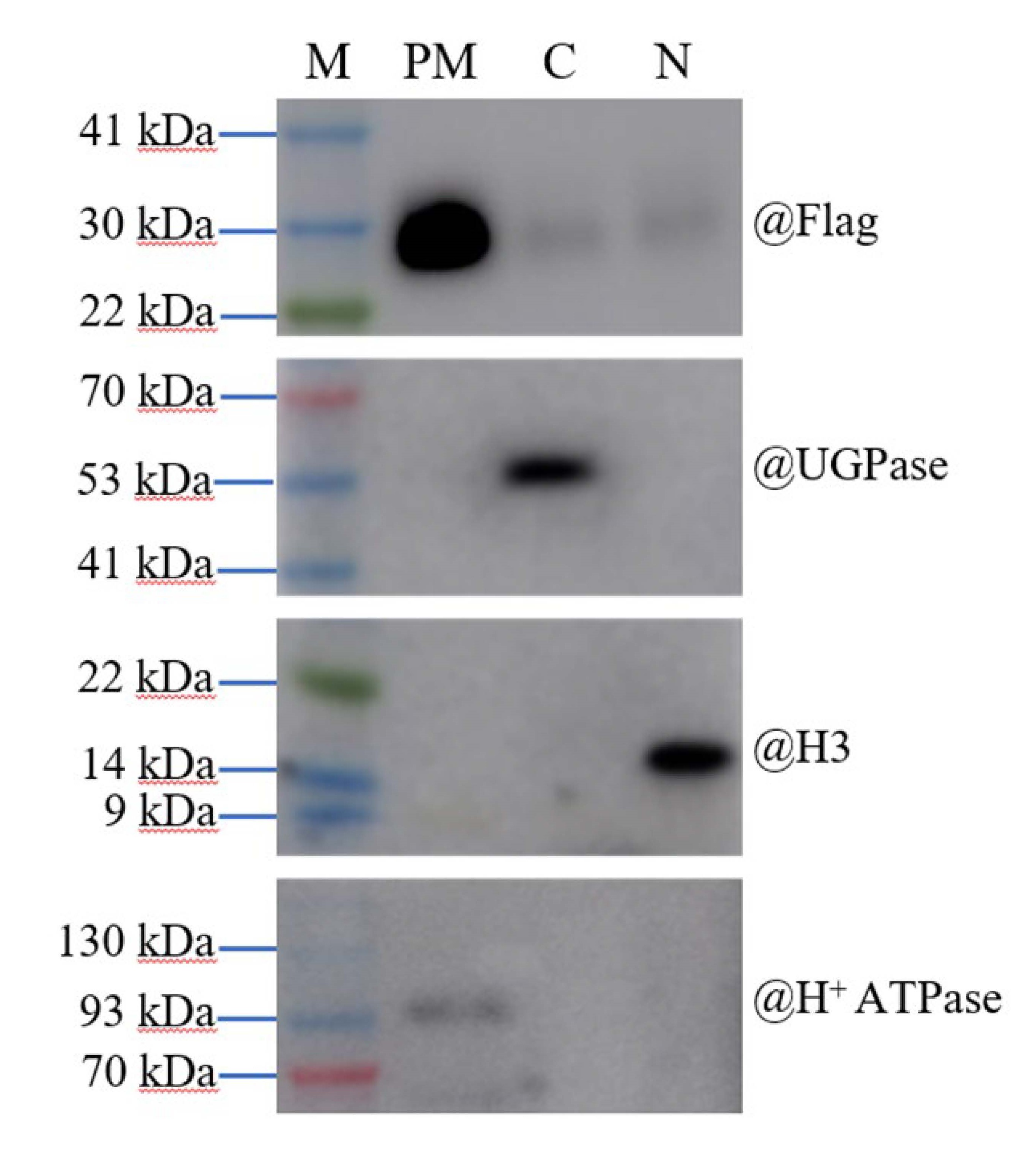

Supplement: Supplementary file 2 — Figure S2 Subcellular fractionation assay of SRBSDV P9‐2 expressed in Nicotiana benthamiana leaves. M, marker for molecular weight of proteins; PM, plasma membrane fraction; C, cytoplasm fraction; N, nuclear fraction [file MPP-24-59-s011.jpg]

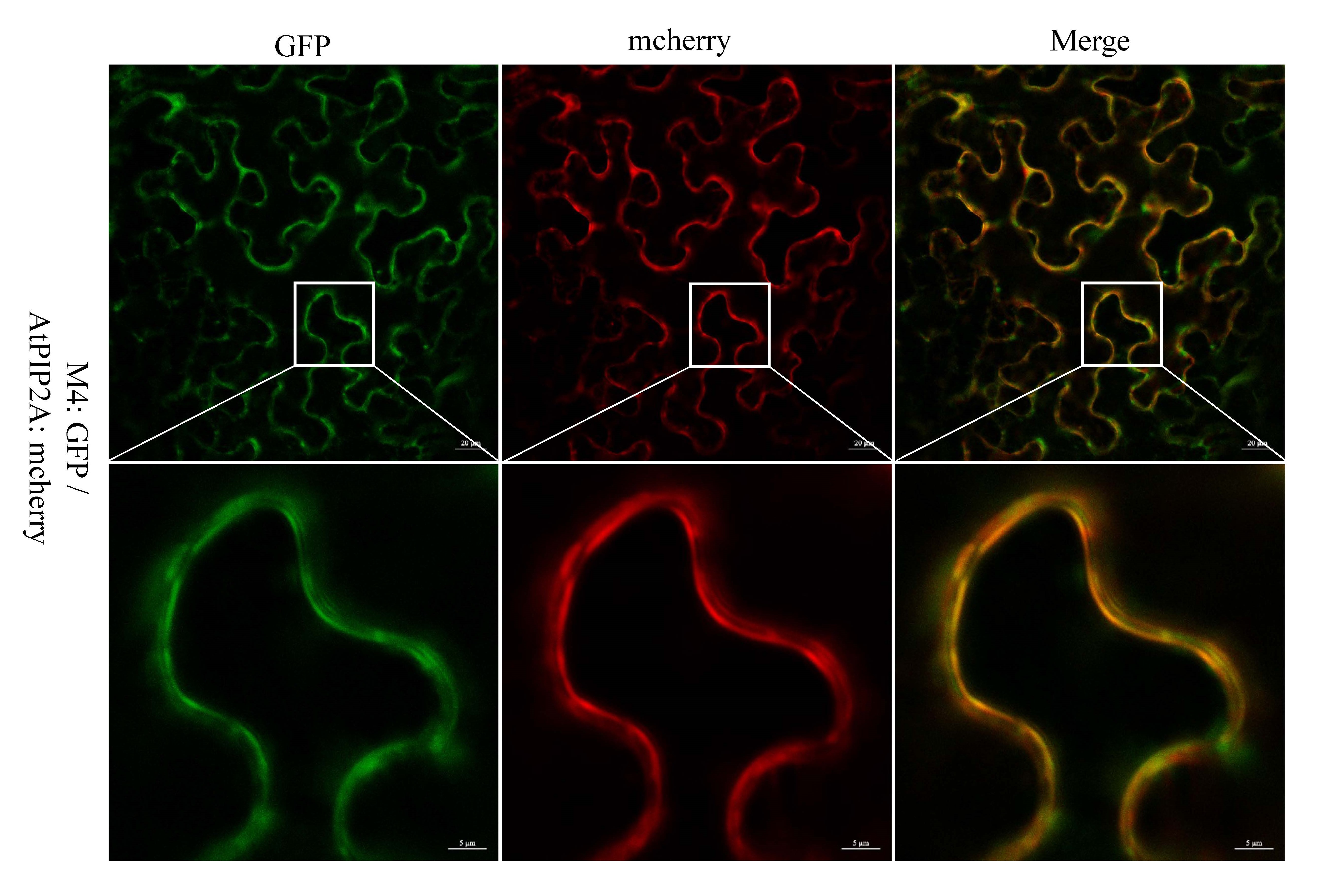

Supplement: Supplementary file 3 — Figure S3 P9‐2M4 mutant retaining the entire two transmembrane helices but deleting N‐ or C‐ terminal parts was colocalized with AtpPIP2A, a marker labelling plasma membrane in plant cells [file MPP-24-59-s003.jpg]

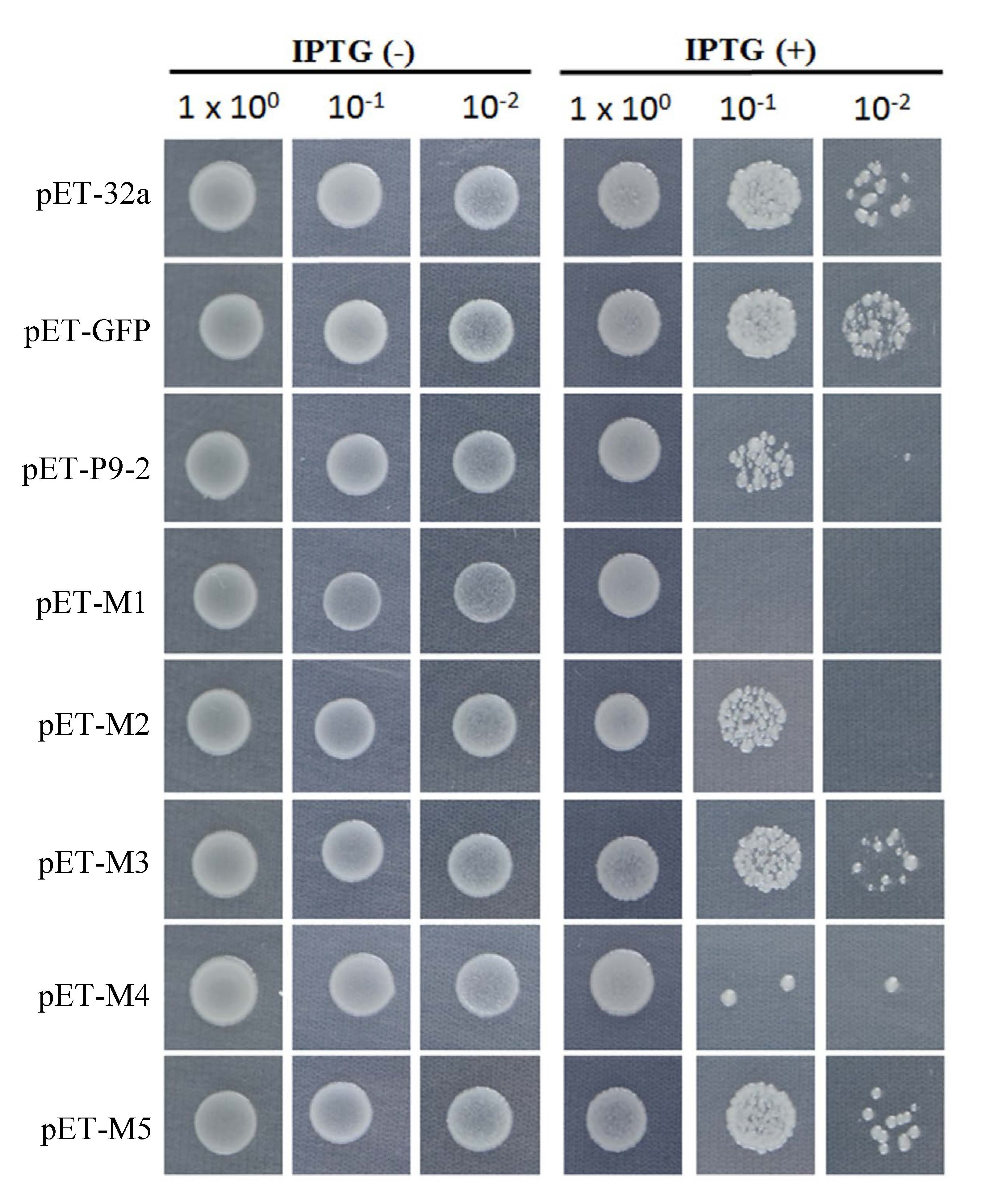

Supplement: Supplementary file 4 — Figure S4 The ability of SRBSDV P9‐2 and its mutants to induce cell death in Escherichia coli [file MPP-24-59-s013.jpg]

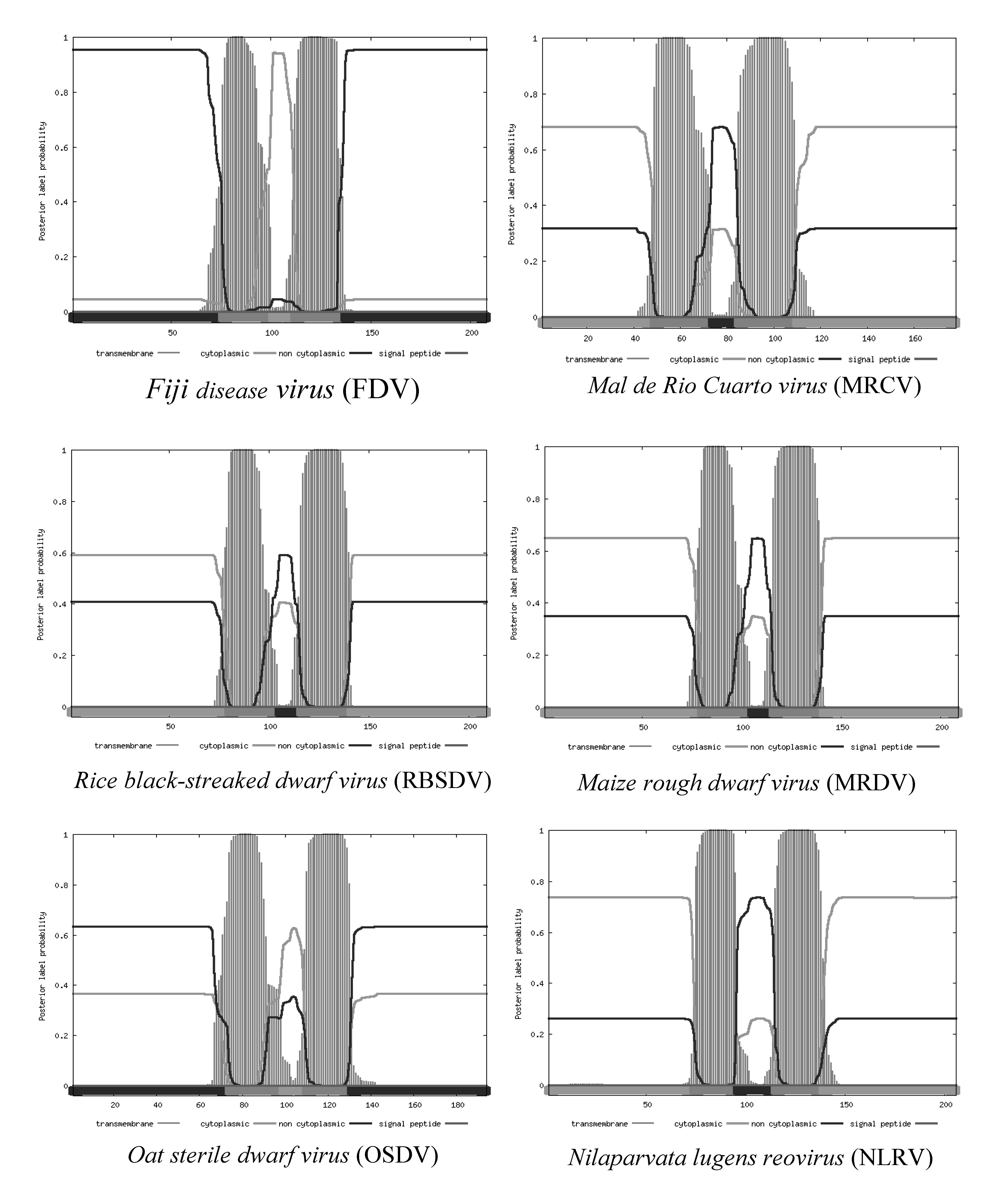

Supplement: Supplementary file 5 — Figure S5 A similar central hydrophobic transmembrane region was predicted in P9‐2 from all other six known fijiviruses, including Fiji disease virus (FDV), Mal de Rio Cuarto virus (MRCV), Maize rough dwarf virus (MRDV), Rice black streaked dwarf virus (RBSDV), Oat sterile dwarf virus (OSDV), and Nilaparvata lugens reovirus (NLRV) [file MPP-24-59-s009.jpg]

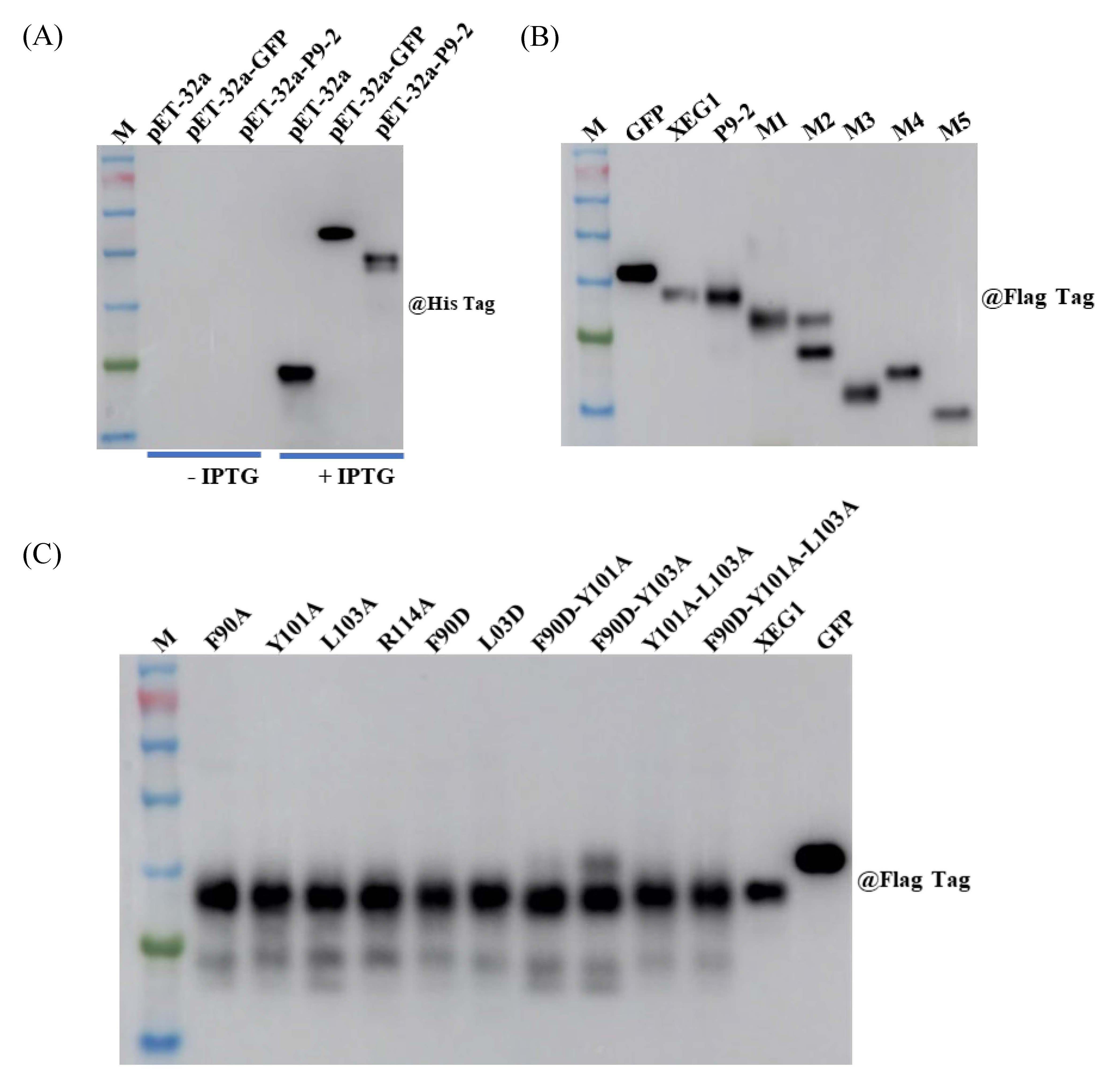

Supplement: Supplementary file 6 — Figure S6 Detection of P9‐2 and its mutant proteins in Escherichia coli (a) and plant cells (b, c) by western blotting assays. (a) Inducible expression of SRBSDV P9‐2 in E. coli. (b) Expression of P9‐2 and its truncated proteins in leaves of Nicotiana benthamiana by the tobacco rattle virus system. (c) Expression of P9‐2 proteins with site‐directed mutations in N. benthamiana leaves. M, marker for molecular weight of proteins [file MPP-24-59-s010.jpg]

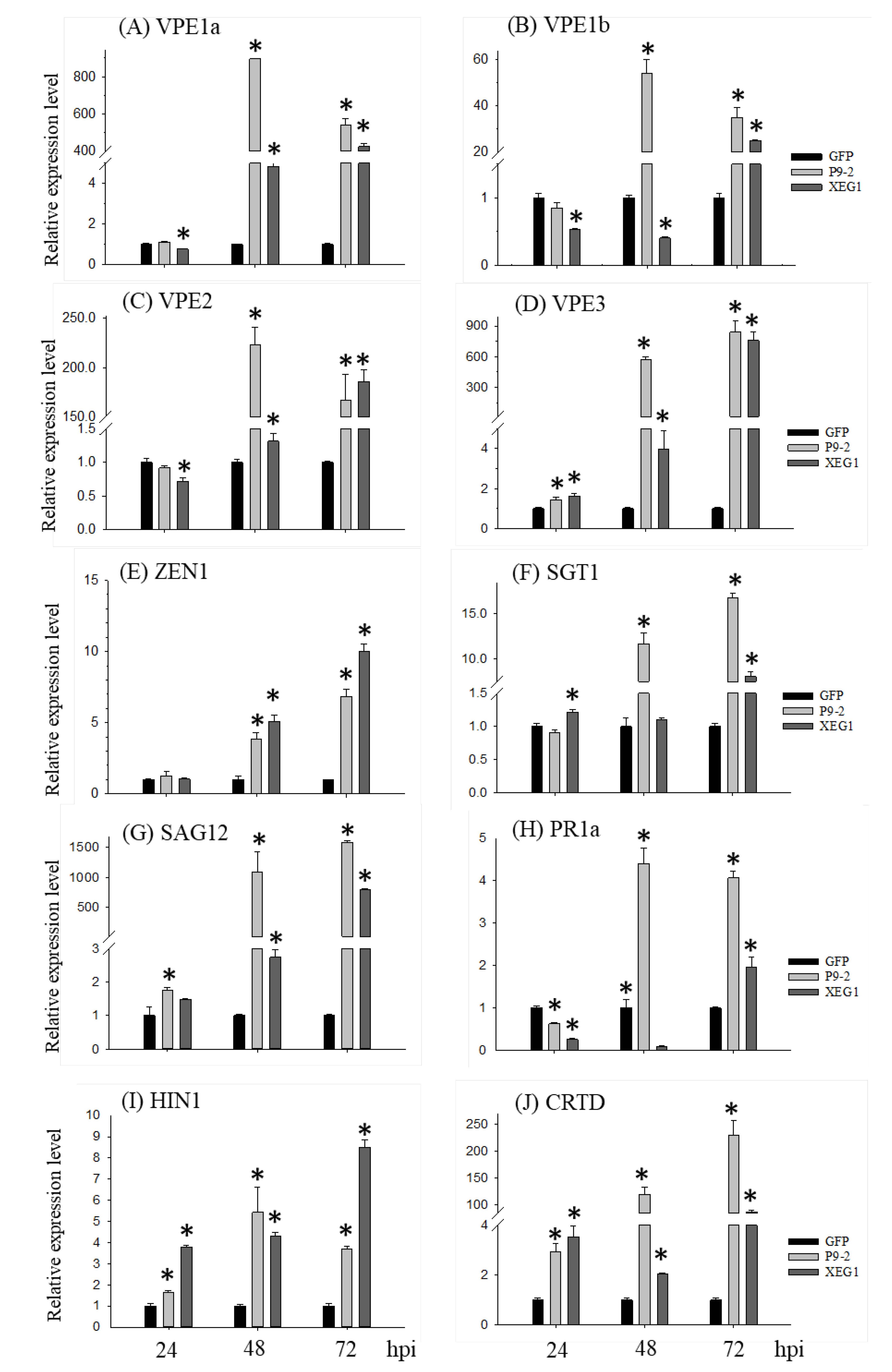

Supplement: Supplementary file 7 — Figure S7 Relative expression level of 10 marker genes for programmed cell death (PCD) in Nicotiana benthamiana leaves agroinfiltrated with TRV‐GFP, ‐P9‐2 or ‐XEG1 plasmids. (a–d) Four vacuolar processing enzyme (VPE) genes, VPE1a, VPE1b, VPE2, and VPE3. (e) Zinnia endonuclease 1 (ZEN1). (f) Suppressor of G2 allele of Skp1 (SGT1). (g) Senescence‐associated gene 12 (SAG12). (h) Pathogenesis‐related 1a (PR1a). (i) Harpin‐induced gene 1 (HIN1). (j) Cys‐rich and transmembrane domain‐containing protein A‐like gene (CRTD) [file MPP-24-59-s005.jpg]

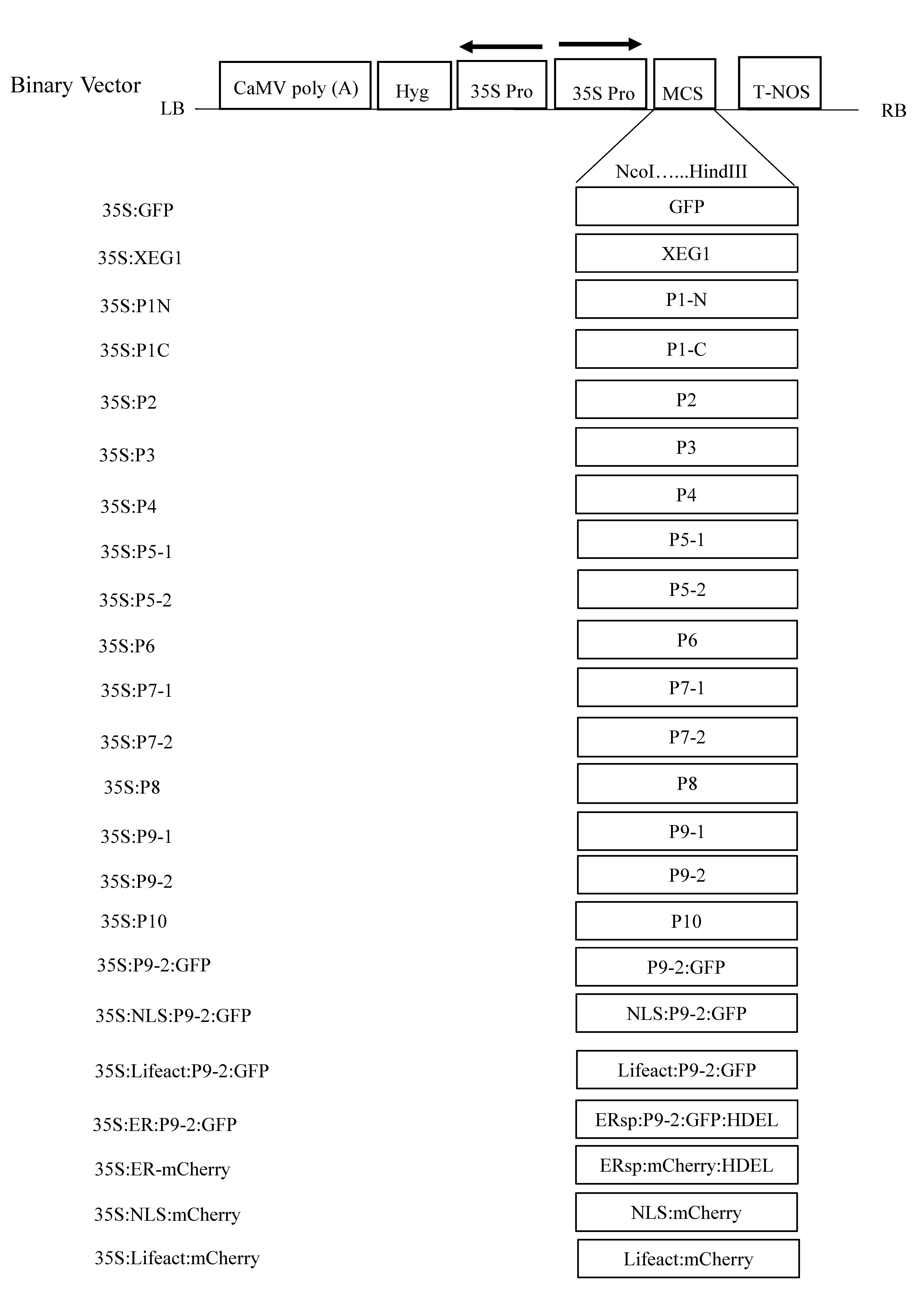

Supplement: Supplementary file 8 — Figure S8 Schematic diagram of vector construction for transient expression or subcellular localization assays [file MPP-24-59-s001.jpg]

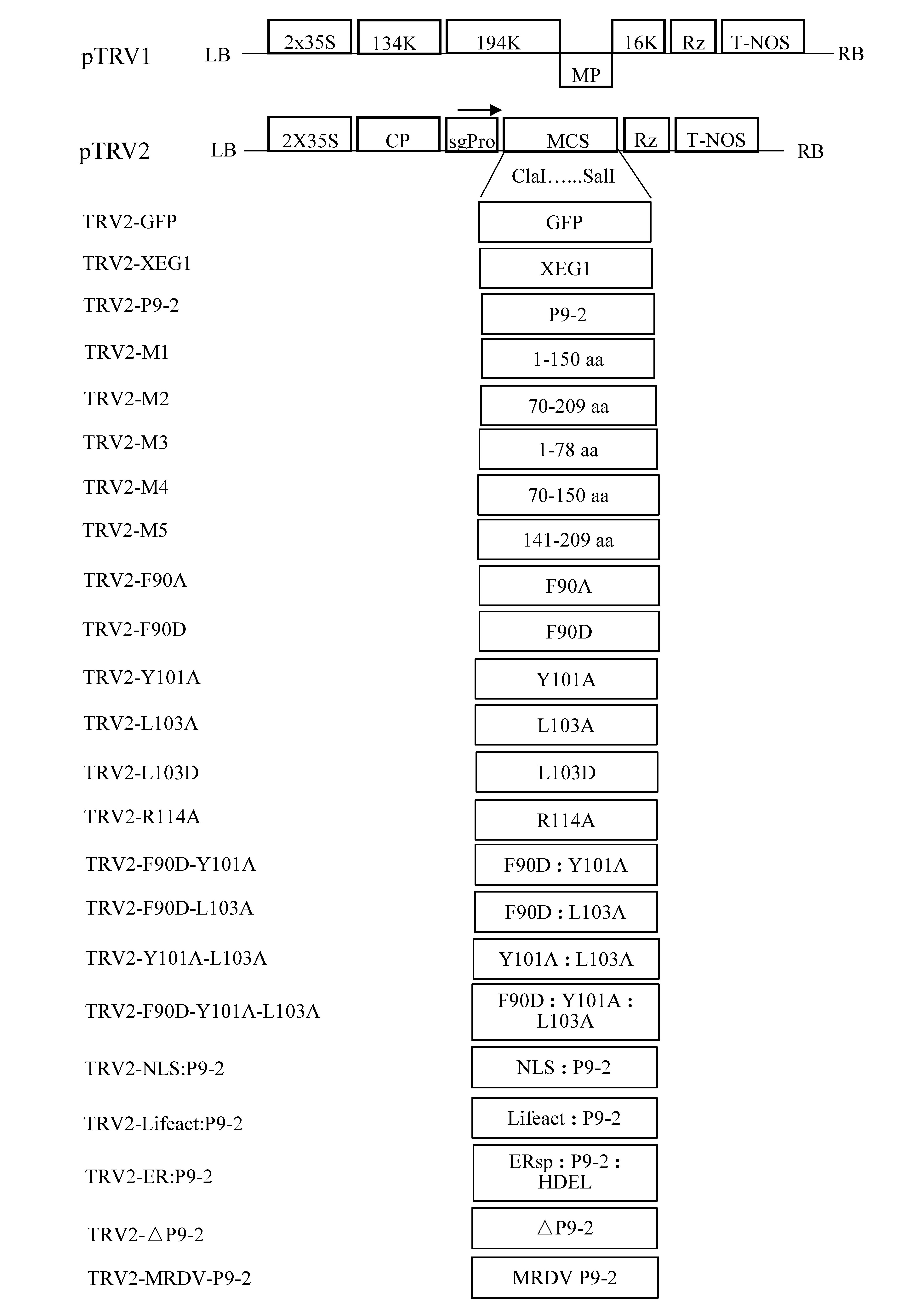

Supplement: Supplementary file 9 — Figure S9 Schematic diagram of vector construction from tobacco rattle virus‐based expression system [file MPP-24-59-s006.jpg]
