# Supplementary material for: A fijiviral nonstructural protein triggers cell death in plant and bacterial cells via its transmembrane domain
Source: Mol Plant Pathol. 2022 Oct 28;24(1):59–70. doi: 10.1111/mpp.13277 (PMC9742498; doi:10.1111/mpp.13277)
Supplement: Supplementary file 10 — Table S1 Primer pairs used for the construction of transient expression in plant [file MPP-24-59-s007.docx]

Table S1 Primer pairs used for the constructions of transient expression in plant.

| **Primer name** | **Sequences (5’-3’)** | **Template** | **Construction** |
| --- | --- | --- | --- |
| P1N-F | GGACTCTTGACCATGGTGGAACGAAAGTTCAGTAGAT | S1 cDNA | 35S:P1N |
| P1N-R | CGACTCTAGAGGATCTCAAAATAATGTAAGGTTAGGAATAG |  |  |
| P1C-F | GGACTCTTGACCATGAAAAATTCTGGAGACATGAAGGG | S1 cDNA | 35S:P1C |
| P1C-R | CGACTCTAGAGGATCTCAGTCGAACTCAAGAGTAAGCATA |  |  |
| P2-F | GGACTCTTGACCATGAATTTGGAGGAGAACCGTAAAA | S2 cDNA | 35S:P2 |
| P2-R | CGACTCTAGAGGATCTCAAATTTGAGATGAAACTCTGATA |  |  |
| P3-F | GGACTCTTGACCATGGATCCAGTACAAGTCATCAAAT | S3 cDNA | 35S:P3 |
| P3-R | CGACTCTAGAGGATCTCAAAATCTCAAAATCTCTGGATGA |  |  |
| P4-F | GGACTCTTGACCATGTTGAAAGTAAATGTGCAGGCCC | S4 cDNA | 35S:P4 |
| P4-R | CGACTCTAGAGGATCTCATTTTTTGGGATGAATTAAGAAA |  |  |
| P5-1-F | GGACTCTTGACCATGACATATTTGAAAGTGAAGATGA | S5 cDNA | 35S:P5-1 |
| P5-1-R | CGACTCTAGAGGATCTCATCGCGCTGTAGTTGGTTGAAGT |  |  |
| P5-2-F | GGACTCTTGACCATGTCTATGACGCGTTGTCCTATTG | S5 cDNA | 35S:P5-2 |
| P5-2-R | CGACTCTAGAGGATCTCAAACATGAAGTATAACTCCTAGC |  |  |
| P6-F | GGACTCTTGACCATGTCTACCAACCTCACGAACATAG | S6 cDNA | 35S:P6 |
| P6-R | CGACTCTAGAGGATCTCACTCTGAAATAAGTTGCCACAAA |  |  |
| P7-1-F | GGACTCTTGACCATGGATAGACCTGCTCGAG | S7 cDNA | 35S:P7-1 |
| P7-1-R | CGACTCTAGAGGATCTCAAGATGATGGAGATTCA |  |  |
| P7-2-F | GGACTCTTGACCATGAATTACAACGATGCCAATG | S7 cDNA | 35S:P7-2 |
| P7-2-R | CGACTCTAGAGGATCTCACAAATTCAGAATGTTTTTC |  |  |
| P8-F | GGACTCTTGACCATGATTGGCACCTATGATGAC | S8 cDNA | 35S:P8 |
| P8-R | CGACTCTAGAGGATCTCAGCAAAGAATAGACACAG |  |  |
| P9-1-F | GGACTCTTGACCATGGCAGACCTAGAGCGTAG | S9 cDNA | 35S:P9-1 |
| P9-1-R | CGACTCTAGAGGATCTCAAACGTCCAATTTAAGTG |  |  |
| P9-2-F | GGACTCTTGACCATGAACCCACAGTCTTCAGT | S9 cDNA | 35S:P9-2 |
| P-2-R | CGACTCTAGAGGATCTCAGTGAAACAAAGTATAATTT |  |  |
| P10-F | GGACTCTTGACCATGGCTGACATAAGACTTGAC | S10 cDNA | 35S:P10 |
| P10-R | CGACTCTAGAGGATCTCATCTGGTGACTTTATTTAAC |  |  |
| GFP-F | GGACTCTTGACCATGGTGAGCAAGGGCGAGGAGC | GFP Plasmid | 35S:GFP |
| GFP-R | CGACTCTAGAGGATCTCACTTGTACAGCTCGTCCATG |  |  |
| XEG1-F | GGACTCTTGACCATGAAGGGATTCTTCGCCGG | XEG1 Plasmid | 35S:XEG1 |
| XEG1-R | CGACTCTAGAGGATCTCAGTTGACCGCAGCCGAGAAC |  |  |
