# Supplementary material for: A fijiviral nonstructural protein triggers cell death in plant and bacterial cells via its transmembrane domain
Source: Mol Plant Pathol. 2022 Oct 28;24(1):59–70. doi: 10.1111/mpp.13277 (PMC9742498; doi:10.1111/mpp.13277)
Supplement: Supplementary file 11 — Table S2 Primer pairs for local and/or systemic expression from the tobacco rattle virus vector in this study [file MPP-24-59-s004.docx]

Table S2 Primer pairs for local and/or systemic expression from TRV vector in this study

| **Primer name** | **Sequences (5’-3’)** | **Template** | **Construction** |
| --- | --- | --- | --- |
| TRV-9-2-F | CTAGCGGATCCATCGATATGAACCCACAGTCTTCAGT | 35S:P9-2 plasmid | TRV2-P9-2 |
| TRV-9-2-R | GGGTACCCGCGTCGACTCAGTGAAACAAAGTATAATTT |  |  |
| TRV-GFP-F | CTAGCGGATCCATCGATATGGTGTCTAAGGGTGAGGAG | GFP plasmid | TRV2-GFP |
| TRV-GFP-R | GGGTACCCGCGTCGACTCACTTGTAAAGCTCGTCCATAC |  |  |
| TRV-XEG1-F | CTAGCGGATCCATCGATATGAAGGGATTCTTCGCCGG | XEG1 plasmid | TRV2-XEG1 |
| TRV-XEG1-R | GGGTACCCGCGTCGACTCAGTTGACCGCAGCCGAGAAC |  |  |
| TRV-M1-F | CTAGCGGATCCATCGATTGAAACCCACAGTCTTCAGT | 35S:P9-2 plasmid | TRV2-△P9-2 |
| TRV-9-2-R | GGGTACCCGCGTCGACTCAGTGAAACAAAGTATAATTT |  |  |
| TRV-MRDV-P9-2-F | CTAGCGGATCCATCGATATGAATCCACAATCTTCAGTG | MRDV P9-2 plasmid | TRV2-MRDV-P9-2 |
| TRV-MRDV-P9-2-R | GGGTACCCGCGTCGACTCAATTAAAAAGAGTATAATTTAC |  |  |
| TRV-9-2-F | CTAGCGGATCCATCGATATGAACCCACAGTCTTCAGT | 35S:P9-2 plasmid | TRV2-M1 |
| TRV-9-2-M1-R | GGGTACCCGCGTCGACTCAAGAATGATATACAGCAAGGA |  |  |
| TRV-9-2-M2-F | CTAGCGGATCCATCGATATGATAATGTTAATGATTTTTAG | 35S:P9-2 plasmid | TRV2-M2 |
| TRV-9-2-R | GGGTACCCGCGTCGACTCAGTGAAACAAAGTATAATTT |  |  |
| TRV-9-2-F | CTAGCGGATCCATCGATATGAACCCACAGTCTTCAGT | 35S:P9-2 plasmid | TRV2-M3 |
| TRV-9-2-M3-R | GGGTACCCGCGTCGACTCAACCAACTTTACCGTATTTTC |  |  |
| TRV-9-2-M2-F | CTAGCGGATCCATCGATATGATAATGTTAATGATTTTTAG | 35S:P9-2 plasmid | TRV2-M4 |
| TRV-9-2-M1-R | GGGTACCCGCGTCGACTCAAGAATGATATACAGCAAGGA |  |  |
| TRV-9-2-M5-F | CTAGCGGATCCATCGATATGGGTGATCAAATCCTTGCTG | 35S:P9-2 plasmid | TRV2-M5 |
| TRV-9-2-R | GGGTACCCGCGTCGACTCAGTGAAACAAAGTATAATTT |  |  |
| TRV-NLS-9-2-F | CTAGCGGATCCATCGATATGGCTCCTAAGAAGAAGCGGAAGGTTGGTATTCACGGGGTGCCTGCGGCTAACCCACAGTCTTCAGT | 35S:P9-2 plasmid | TRV2-NLS:P9-2 |
| TRV-9-2-R | GGGTACCCGCGTCGACTCAGTGAAACAAAGTATAATTT |  |  |
| TRV-Lifeact-9-2-F | CTAGCGGATCCATCGATATGGGTGTTGCTGATCTTATTAAGAAGTTTGAATCTATTTCTAAGGAAGAAAACCCACAGTCTTCAGT | 35S:P9-2 plasmid | TRV2-Lifeact:P9-2 |
| TRV-9-2-R | GGGTACCCGCGTCGACTCAGTGAAACAAAGTATAATTT |  |  |
| TRV-ER-9-2-F | CTAGCGGATCCATCGATATGAAGGTACAGGAGGGTTTGTTCGTGGTGGCTGTTTTCTACCTTGCTTATACGCAGCTAGTCAAGGGGCAACCTCGCAAGGAGTGCAACCCACAGTCTTCAGT | 35S:P9-2 plasmid | TRV2-ER:P9-2 |
| TRV-9-2-HDEL-R | GGGTACCCGCGTCGACTCACAGCTCGTCATGGTGAAACAAAGTATAATTT |  |  |
| TRV-9-2-F | CTAGCGGATCCATCGATATGAACCCACAGTCTTCAGT | 35S:P9-2 plasmid | TRV2-F90A |
| TRV-F90A-R | GAAAAGAAAGCTAAAAATCATTAAC |  |  |
| TRV-F90A-F | TGATTTTTAGCTTTCTTTTCGCTGGGATTTTTAAATTAACG | 35S:P9-2 plasmid， |  |
| TRV-9-2-R | GGGTACCCGCGTCGACTCAGTGAAACAAAGTATAATTT |  |  |
| ClaI-TRV-9-2-F | CTAGCGGATCCATCGATATGAACCCACAGTCTTCAGT | 35S:P9-2 plasmid | TRV2-F90D |
| TRV-F90D-R | GAAAAGAAAGCTAAAAATCATTAAC |  |  |
| TRV-F90D-F | TGATTTTTAGCTTTCTTTTCGATGGGATTTTTAAATTAACG | 35S:P9-2 plasmid |  |
| TRV-9-2-R | GGGTACCCGCGTCGACTCAGTGAAACAAAGTATAATTT |  |  |
| TRV-9-2-F | CTAGCGGATCCATCGATATGAACCCACAGTCTTCAGT | 35S:P9-2 plasmid | TRV2-Y101A |
| TRV-Y101A-R | CATTTTAAGCGTTAATTTAAAAATC |  |  |
| TRV-Y101A-F | TTAAATTAACGCTTAAAATGTTTGCTCATCTTTTTCGGTGTGTATGTTGT | 35S:P9-2 plasmid |  |
| TRV-9-2-R | GGGTACCCGCGTCGACTCAGTGAAACAAAGTATAATTT |  |  |
| TRV-9-2-F | CTAGCGGATCCATCGATATGAACCCACAGTCTTCAGT | 35S:P9-2 plasmid | TRV2-L103A |
| TRV-L103A-R | CATTTTAAGCGTTAATTTAAAAATC |  |  |
| TRV-L103A-F | TTAAATTAACGCTTAAAATGTTTTATCATGCTTTTCGGTGTGTATGTTGT | 35S:P9-2 plasmid |  |
| TRV-9-2-R | GGGTACCCGCGTCGACTCAGTGAAACAAAGTATAATTT |  |  |
| TRV-9-2-F | CTAGCGGATCCATCGATATGAACCCACAGTCTTCAGT | 35S:P9-2 plasmid | TRV2-L103D |
| TRV-L103D-R | CATTTTAAGCGTTAATTTAAAAATC |  |  |
| TRV-L103D-F | TTAAATTAACGCTTAAAATGTTTTATCATGATTTTCGGTGTGTATGTTGT | 35S:P9-2 plasmid |  |
| TRV-9-2-R | GGGTACCCGCGTCGACTCAGTGAAACAAAGTATAATTT |  |  |
| TRV-9-2-F | CTAGCGGATCCATCGATATGAACCCACAGTCTTCAGT | 35S:P9-2 plasmid | TRV2-R114A |
| TRV-R114A-R | AATTAAAGGATTACAACATACAC |  |  |
| TRV-R114A-F | GTATGTTGTAATCCTTTAATTGCTGGAATTTTTAGTGTTGTTTG | 35S:P9-2 plasmid |  |
| TRV-9-2-R | GGGTACCCGCGTCGACTCAGTGAAACAAAGTATAATTT |  |  |
| TRV-9-2-F | CTAGCGGATCCATCGATATGAACCCACAGTCTTCAGT | 35S:P9-2 plasmid | TRV2-Y101A-L103A |
| TRV-Y101-L103-R | CATTTTAAGCGTTAATTTAAAAATC |  |  |
| TRV-Y101A-L103A-F | TTAAATTAACGCTTAAAATGTTTGCTCATGCTTTTCGGTGTGTATGTTGT | 35S:P9-2 plasmid |  |
| TRV-9-2-R | GGGTACCCGCGTCGACTCAGTGAAACAAAGTATAATTT |  |  |
| TRV-9-2-F | CTAGCGGATCCATCGATATGAACCCACAGTCTTCAGT | TRV2:△F90D plasmid | TRV2-F90D-Y101A |
| TRV-Y101-L103-R | CATTTTAAGCGTTAATTTAAAAATC |  |  |
| TRV-Y101A-F | TTAAATTAACGCTTAAAATGTTTGCTCATCTTTTTCGGTGTGTATGTTGT | 35S:P9-2 plasmid |  |
| TRV-9-2-R | GGGTACCCGCGTCGACTCAGTGAAACAAAGTATAATTT |  |  |
| TRV-9-2-F | CTAGCGGATCCATCGATATGAACCCACAGTCTTCAGT | TRV2-△F90D plasmid | TRV2-F90D-L103A |
| TRV-Y101-L103-R | CATTTTAAGCGTTAATTTAAAAATC |  |  |
| TRV-L103A-F | TTAAATTAACGCTTAAAATGTTTTATCATGCTTTTCGGTGTGTATGTTGT | 35S:P9-2 plasmid |  |
| TRV-9-2-R | GGGTACCCGCGTCGACTCAGTGAAACAAAGTATAATTT |  |  |
| TRV-9-2-F | CTAGCGGATCCATCGATATGAACCCACAGTCTTCAGT | TRV2-△F90D plasmid | TRV2-F90D-Y101A-L103A |
| TRV-Y101-L103-R | CATTTTAAGCGTTAATTTAAAAATC |  |  |
| TRV-Y101A-L103A-F | TTAAATTAACGCTTAAAATGTTTGCTCATGCTTTTCGGTGTGTATGTTGT | 35S:P9-2 plasmid |  |
| TRV-9-2-R | GGGTACCCGCGTCGACTCAGTGAAACAAAGTATAATTT |  |  |
