# Supplementary material for: A fijiviral nonstructural protein triggers cell death in plant and bacterial cells via its transmembrane domain
Source: Mol Plant Pathol. 2022 Oct 28;24(1):59–70. doi: 10.1111/mpp.13277 (PMC9742498; doi:10.1111/mpp.13277)
Supplement: Supplementary file 12 — Table S3 Primer pairs used for the construction of prokaryotic expression in Escherichia coli [file MPP-24-59-s008.docx]

Table S3 Primer pairs used for the constructions of procaryotic expression in *E. coli*

| **Primer names** | **Sequences（5'-3'）** | **Templates** | **Constructions** |
| --- | --- | --- | --- |
| pET32a-p9-2-F | TATCGGATCCGAATTCATGAACCCACAGTCTTCAGT | 35S:P9-2 plasmid | pET-p9-2 |
| pET32a-p9-2-R | GGTGGTGGTGCTCGAGGTGAAACAAAGTATAATTT |  |  |
| pET32a-GFP-F | TATCGGATCCGAATTCATGGTGAGCAAGGGCGAGGAGC | GFP plasmid | pET-GFP |
| pET32a-GFP-R | GGTGGTGGTGCTCGAGCTTGTACAGCTCGTCCATG |  |  |
| pET32a-p9-2-F | TATCGGATCCGAATTCATGAACCCACAGTCTTCAGT | TRV2-M1 plasmid | pET-M1 |
| pET32a-M1-R | GGTGGTGGTGCTCGAGagaatgatatacagcaagga |  |  |
| pET32a-M2-F | TATCGGATCCGAATTCATGataatgttaatgatttttag | TRV2-M2 plasmid | pET-M2 |
| pET32a-p9-2-R | GGTGGTGGTGCTCGAGGTGAAACAAAGTATAATTT |  |  |
| pET32a-p9-2-F | TATCGGATCCGAATTCATGAACCCACAGTCTTCAGT | TRV2-M3 plasmid | pET-M3 |
| pET32a-M3-R | GGTGGTGGTGCTCGAGaccaactttaccgtattttc |  |  |
| pET32a-M2-F | TATCGGATCCGAATTCATGataatgttaatgatttttag | TRV2-M4 plasmid | pET-M4 |
| pET32a-M1-R | GGTGGTGGTGCTCGAGagaatgatatacagcaagga |  |  |
| pET32a-M5-F | TATCGGATCCGAATTCATGggtgatcaaatccttgctg | TRV2-M5 plasmid | pET-M5 |
| pET32a-p9-2-R | GGTGGTGGTGCTCGAGGTGAAACAAAGTATAATTT |  |  |
| pET32a-p9-2-F | TATCGGATCCGAATTCATGAACCCACAGTCTTCAGT | TRV2-△F90D-Y101A-L103A plasmid | pET-F90D-Y101A-L103A |
| pET32a-p9-2-R | GGTGGTGGTGCTCGAGGTGAAACAAAGTATAATTT |  |  |
