# Supplementary material for: A fijiviral nonstructural protein triggers cell death in plant and bacterial cells via its transmembrane domain
Source: Mol Plant Pathol. 2022 Oct 28;24(1):59–70. doi: 10.1111/mpp.13277 (PMC9742498; doi:10.1111/mpp.13277)
Supplement: Supplementary file 13 — Table S4 Primer pairs used for vector construction in assays of subcellular localization [file MPP-24-59-s002.docx]

Table S4 Primer pairs used for vector construction in assays of subcellular localization.

| **Primer name** | **Sequences (5’-3’)** | **Template** | **Construction** |
| --- | --- | --- | --- |
| NLS-mCherry-F | GGACTCTTGACCATGGCTCCTAAGAAGAAGCGGAAGGTTGGTATTCACGGGGTGCCTGCGGCTGTGTCTAAGGGTGAGGAG | mCherry plasmid | 35S:NLS:mCherry |
| mCherry-R | CGACTCTAGAGGATCTCACTTGTAAAGCTCGTCCATAC |  |  |
| Lifeact-mCherry-F | GGACTCTTGACCATGGGTGTTGCTGATCTTATTAAGAAGTTTGAATCTATTTCTAAGGAAGAAGTGTCTAAGGGTGAGGAG | mCherry plasmid | 35S:Lifeact:mCherry |
| mCherry-R | CGACTCTAGAGGATCTCACTTGTAAAGCTCGTCCATAC |  |  |
| ER-mCherry-F | GGACTCTTGACCATGAAGGTACAGGAGGGTTTGTTCGTGGTGGCTGTTTTCTACCTTGCTTATACGCAGCTAGTCAAGGGGCAACCTCGCAAGGAGTGCGTGTCTAAGGGTGAGGAG | mCherry plasmid | 35S:ER:mCherry |
| mCherry-HDEL-R | CGACTCTAGAGGATCTCACAGCTCGTCATGCTTGTAAAGCTCGTCCATAC |  |  |
| PIP2A-F | GGACTCTTGACCATGGCAAAGGATGTGGAAGCCGTTC | AtPIP2A plamsid | 35S:PIP2A:mCherry |
| PIP2A-mCherry-R | CTTGTAAAGCTCGTCCATACGTGAAACAAAGTATAATTT |  |  |
| PIP2A-mCherry-F | AAATTATACTTTGTTTCACGTGTCTAAGGGTGAGGAG | mCherry plasmid |  |
| mCherry-R | CGACTCTAGAGGATCTCACTTGTAAAGCTCGTCCATAC |  |  |
| P9-2-F | GGACTCTTGACCATGAACCCACAGTCTTCAGT | 35S:P9-2 plasmid | 35S:P9-2:GFP |
| P9-2-GFP-R | GCTCCTCGCCCTTGCTCACGTGAAACAAAGTATAATTT |  |  |
| P9-2-GFP-F | AAATTATACTTTGTTTCACGTGAGCAAGGGCGAGGAGC | GFP plasmid |  |
| GFP-R | CGACTCTAGAGGATCTCACTTGTACAGCTCGTCCATG |  |  |
| NLS-P9-2-F | GGACTCTTGACCATGGCTCCTAAGAAGAAGCGGAAGGTTGGTATTCACGGGGTGCCTGCGGCTAACCCACAGTCTTCAGT | 35S:P9-2 plasmid | 35S:NLS:P9-2:GFP |
| P9-2-GFP-R | GCTCCTCGCCCTTGCTCACGTGAAACAAAGTATAATTT |  |  |
| P9-2-GFP-F | AAATTATACTTTGTTTCACGTGAGCAAGGGCGAGGAGC | GFP plasmid |  |
| GFP-R | CGACTCTAGAGGATCTCACTTGTACAGCTCGTCCATG |  |  |
| Lifeact-P9-2-F | GGACTCTTGACCATGGGTGTTGCTGATCTTATTAAGAAGTTTGAATCTATTTCTAAGGAAGAAAACCCACAGTCTTCAGT | 35S:P9-2 plasmid | 35S:Lifeact:P9-2:GFP |
| P9-2-GFP-R | GCTCCTCGCCCTTGCTCACGTGAAACAAAGTATAATTT |  |  |
| P9-2-GFP-F | AAATTATACTTTGTTTCACGTGAGCAAGGGCGAGGAGC | GFP plasmid |  |
| GFP-R | CGACTCTAGAGGATCTCACTTGTACAGCTCGTCCATG |  |  |
| ER-P9-2-F | GGACTCTTGACCATGAAGGTACAGGAGGGTTTGTTCGTGGTGGCTGTTTTCTACCTTGCTTATACGCAGCTAGTCAAGGGGCAACCTCGCAAGGAGTGCAACCCACAGTCTTCAGT | 35S:P9-2 plasmid | 35S:ER:P9-2:GFP |
| P9-2-GFP-R | GCTCCTCGCCCTTGCTCACGTGAAACAAAGTATAATTT |  |  |
| P9-2-GFP-F | AAATTATACTTTGTTTCACGTGAGCAAGGGCGAGGAGC | GFP plasmid |  |
| GFP-HDEL-R | CGACTCTAGAGGATCTCACAGCTCGTCATGCTTGTACAGCTCGTCCATG |  |  |
